# Supplementary figures and images for: In leukemia, knock-down of the death inducer-obliterator gene would inhibit the proliferation of endothelial cells by inhibiting the expression of CDK6 and CCND1
Source: PeerJ. 2022 Feb 1;10:e12832. doi: 10.7717/peerj.12832 (PMC8815367; doi:10.7717/peerj.12832)

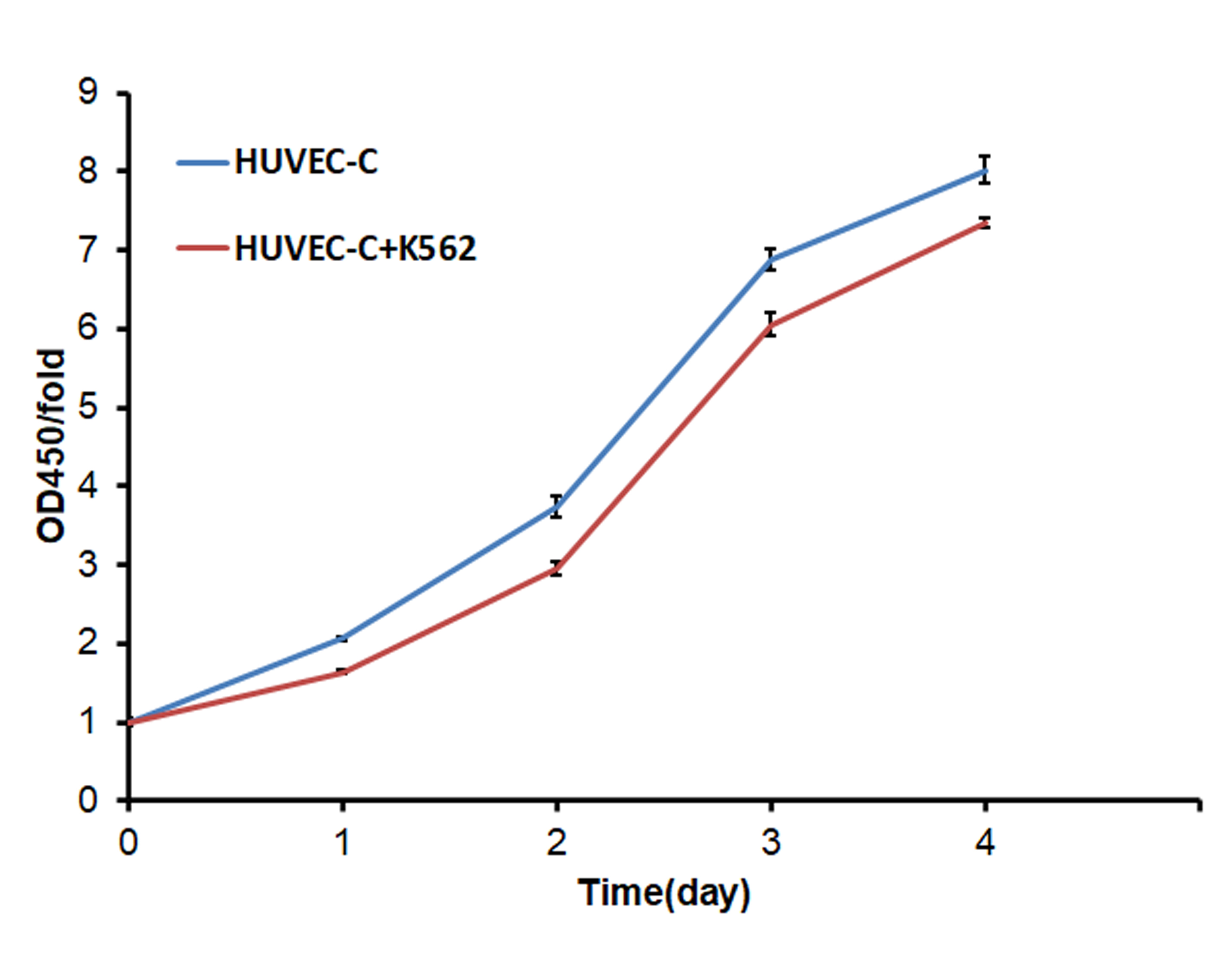

Supplement: Supplemental Information 1 [file peerj-10-12832-s001.jpg]

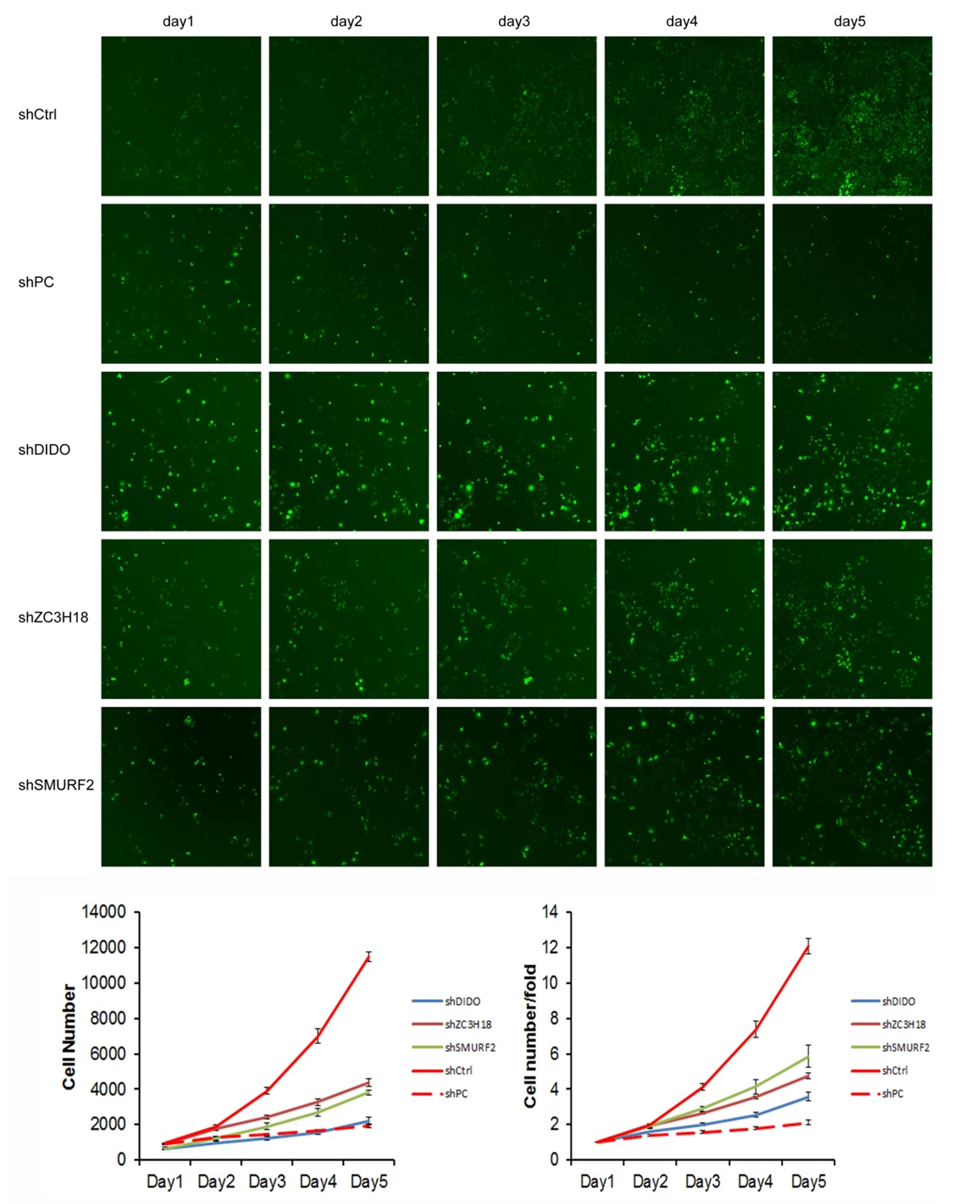

Supplement: Supplemental Information 2 — (A) The Volcano map of the differently expressed probes; (B) The signal pathway histogram shows the enrichment of DEGs in the classical signal pathway [file peerj-10-12832-s002.jpg]

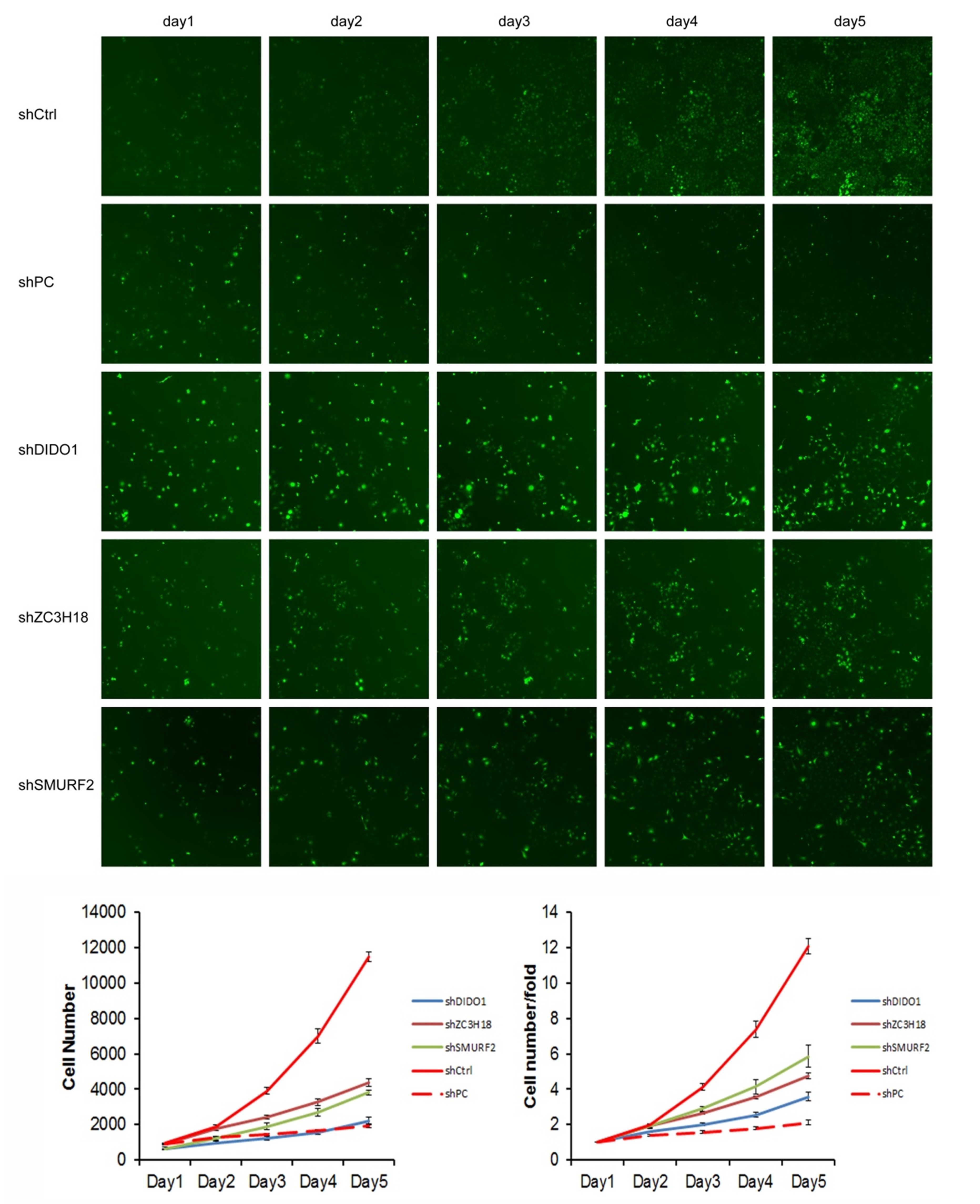

Supplement: Supplemental Information 3 [file peerj-10-12832-s003.jpg]

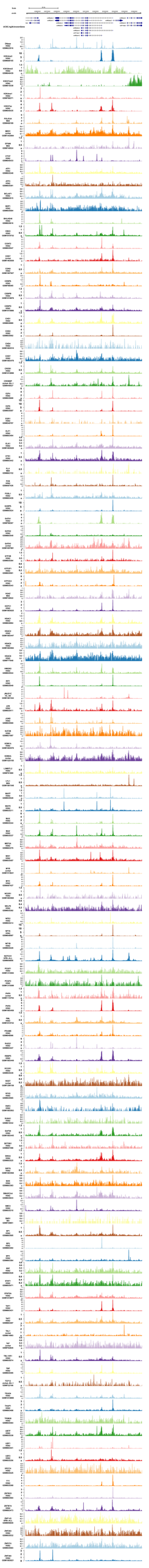

Supplement: Supplemental Information 4 [file peerj-10-12832-s004.pdf]

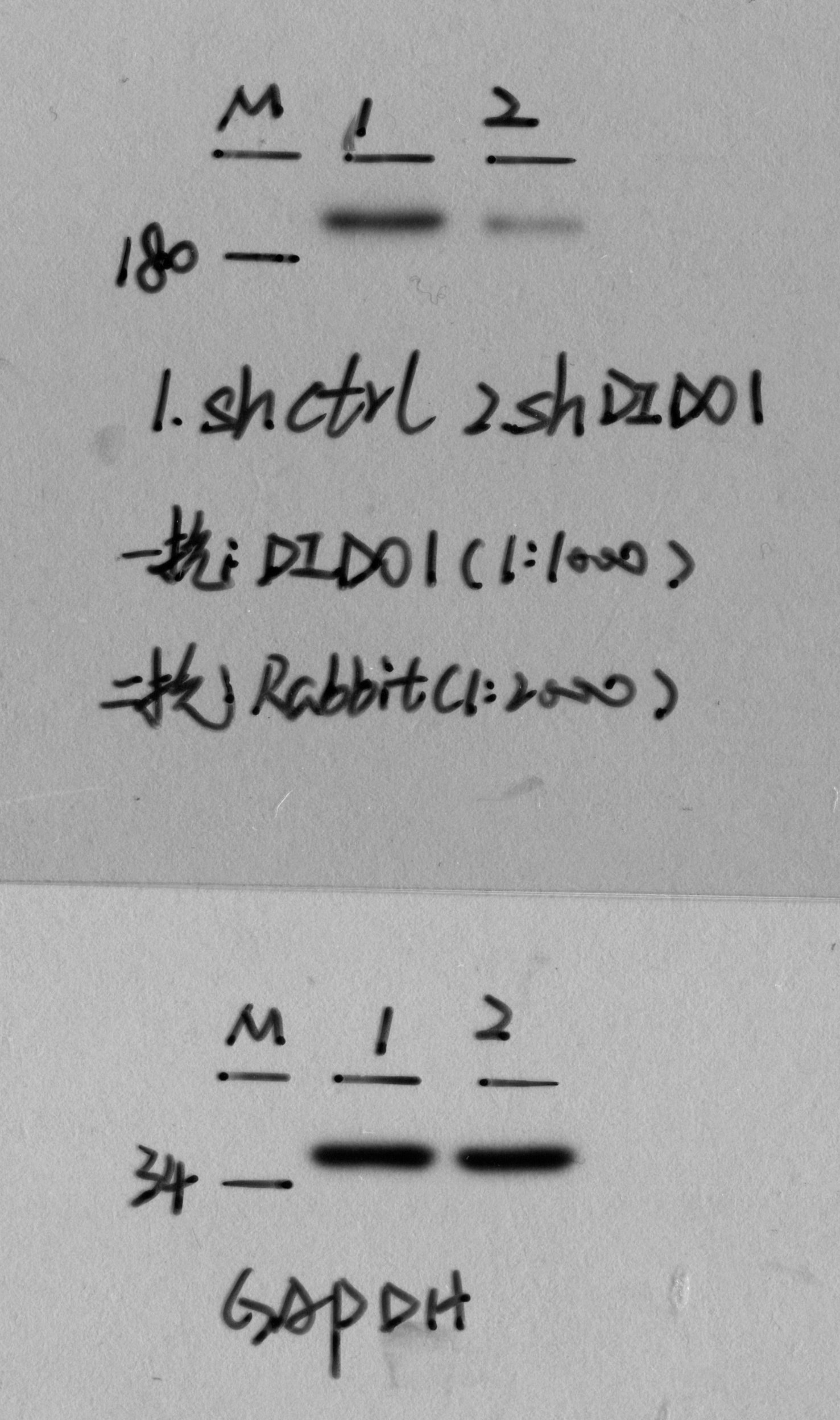

Supplement: Supplemental Information 11 [file peerj-10-12832-s011.zip › Figure1B-original-WB.jpg]

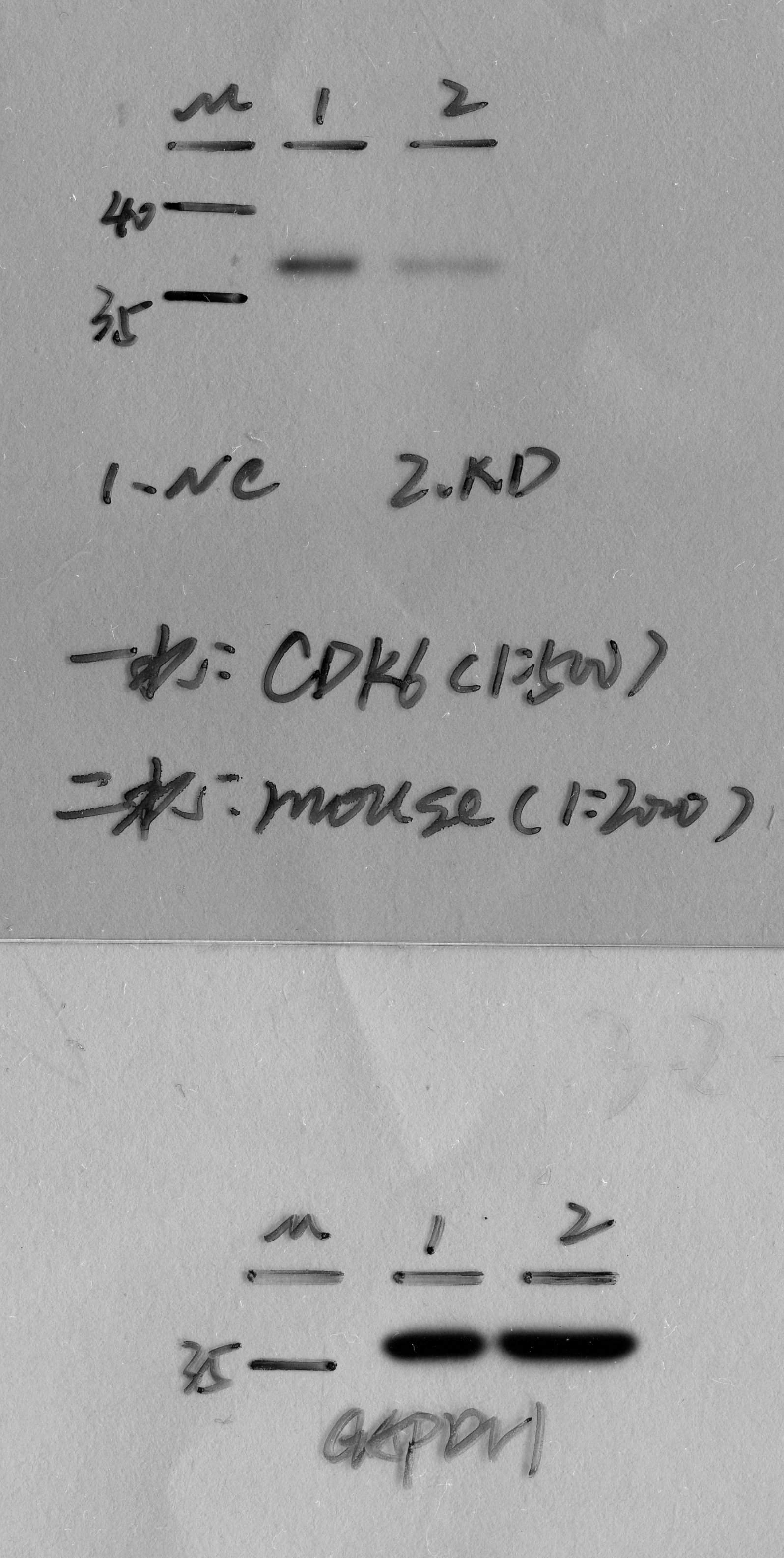

Supplement: Supplemental Information 11 [file peerj-10-12832-s011.zip › Figure2E-CDK6-WB-Original.jpg]

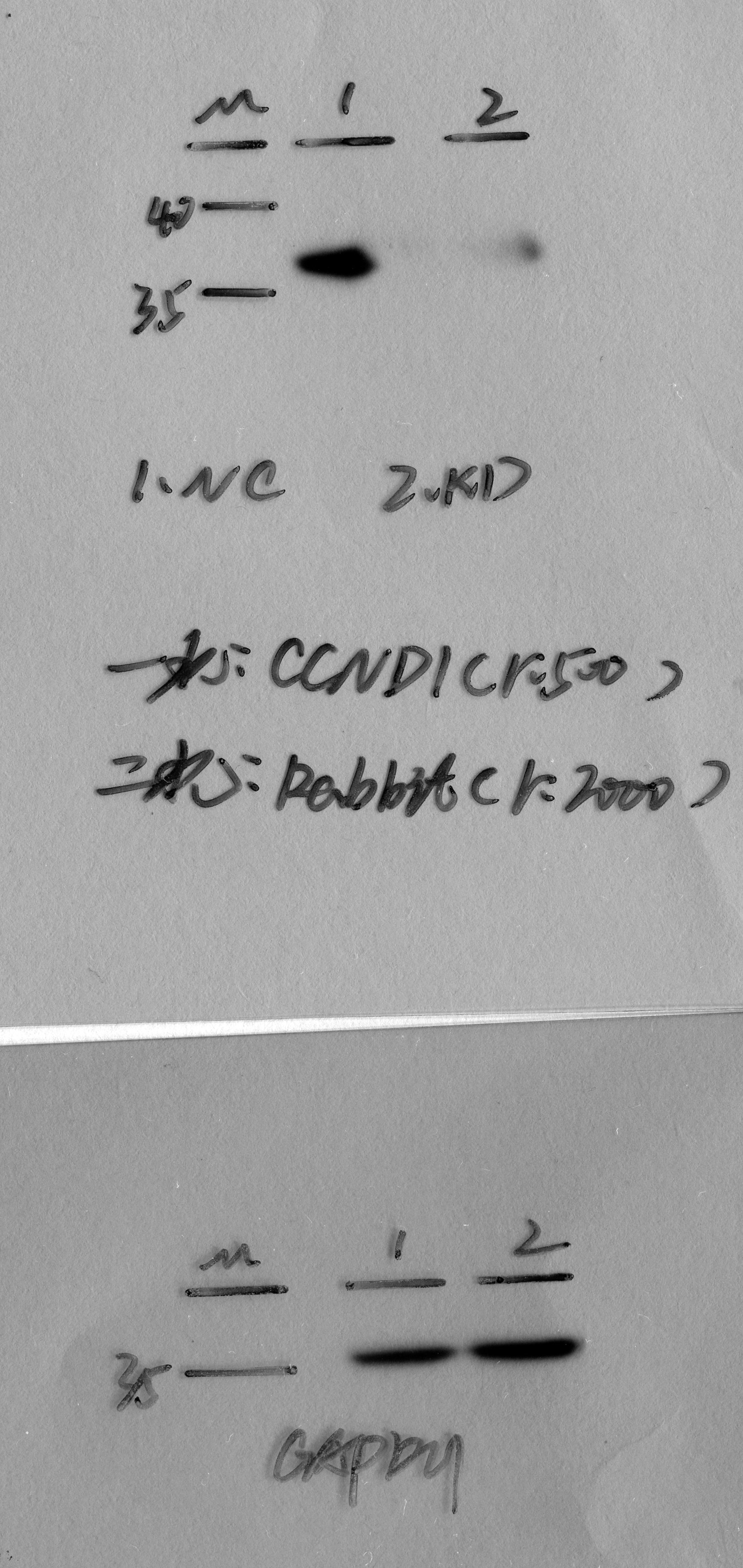

Supplement: Supplemental Information 11 [file peerj-10-12832-s011.zip › Figure2E-CCND1-WB-Original.jpg]

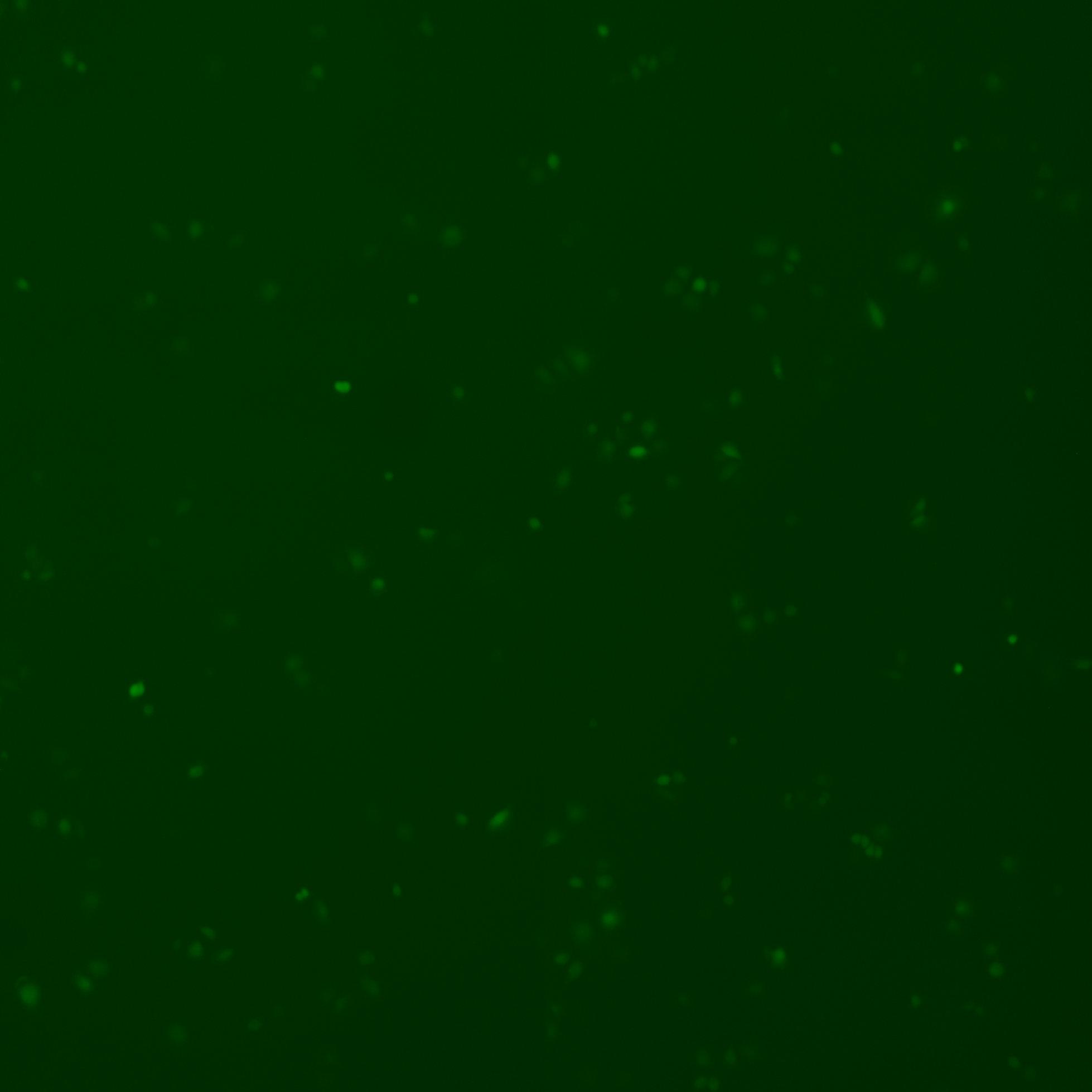

Supplement: Supplemental Information 12 [file peerj-10-12832-s012.zip › Original images 4 cell cultures/Figure1C-shCtrl-day1.jpg]

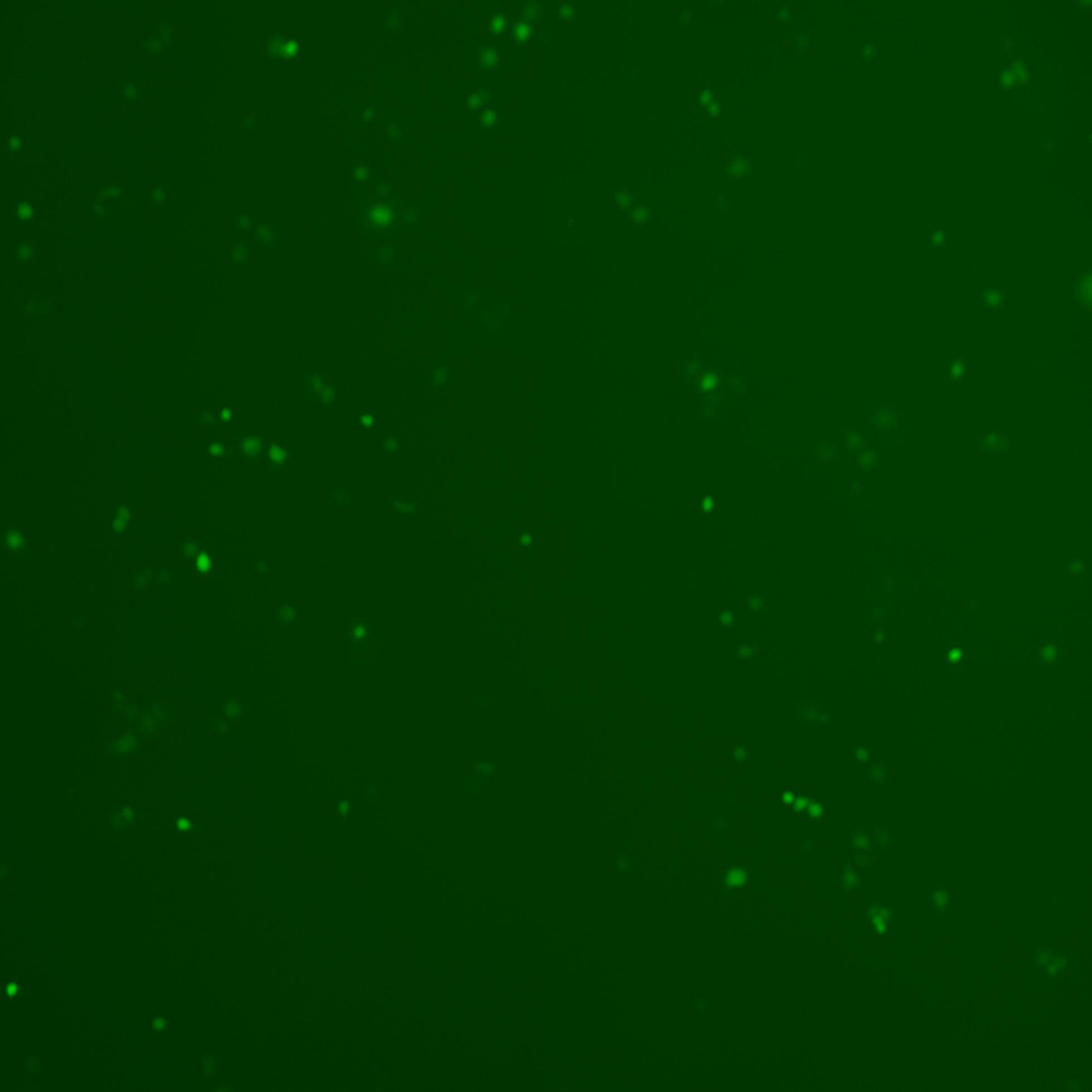

Supplement: Supplemental Information 12 [file peerj-10-12832-s012.zip › Original images 4 cell cultures/Figure1C-shDIDO1-day1.jpg]

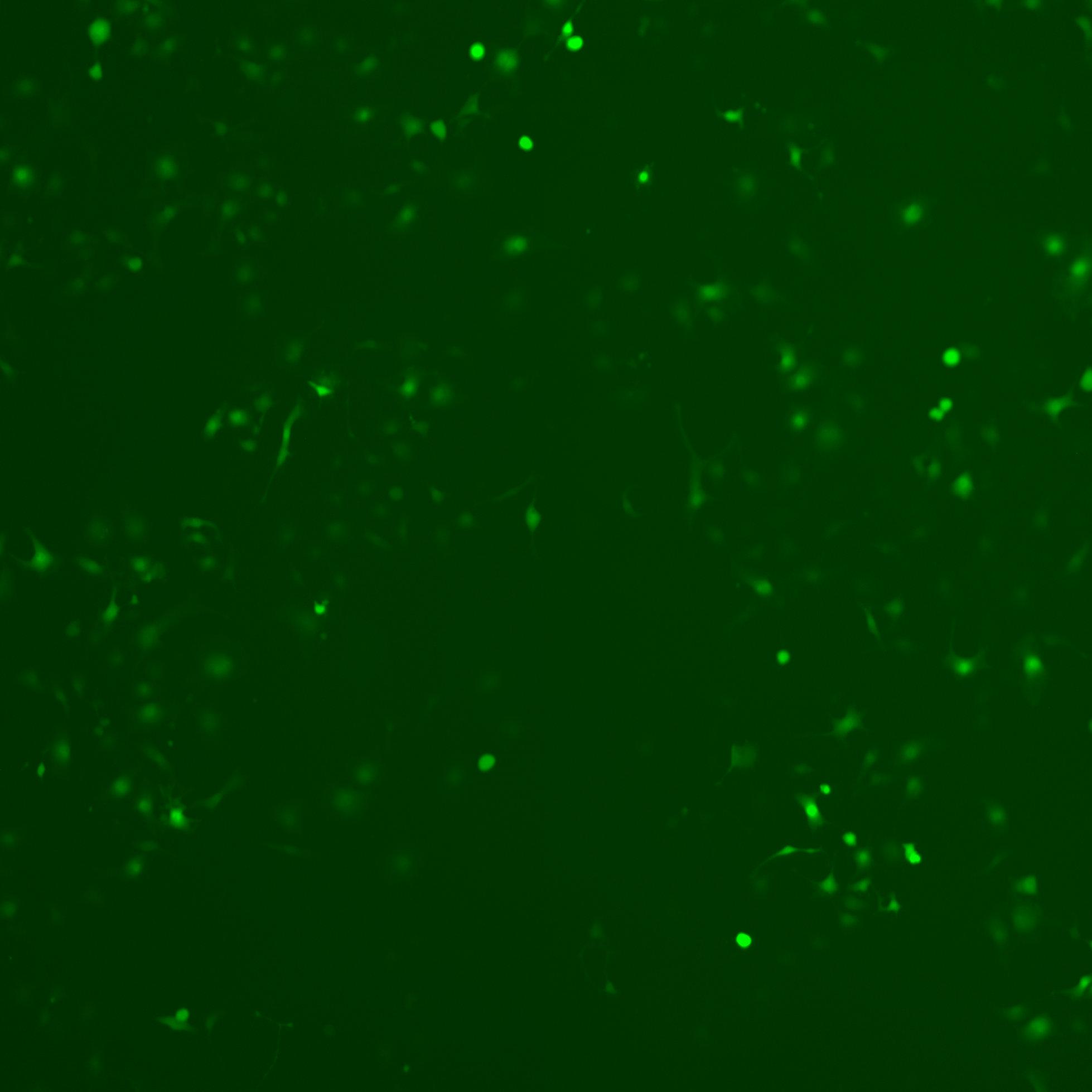

Supplement: Supplemental Information 12 [file peerj-10-12832-s012.zip › Original images 4 cell cultures/Figure1C-shDIDO1-day3.jpg]

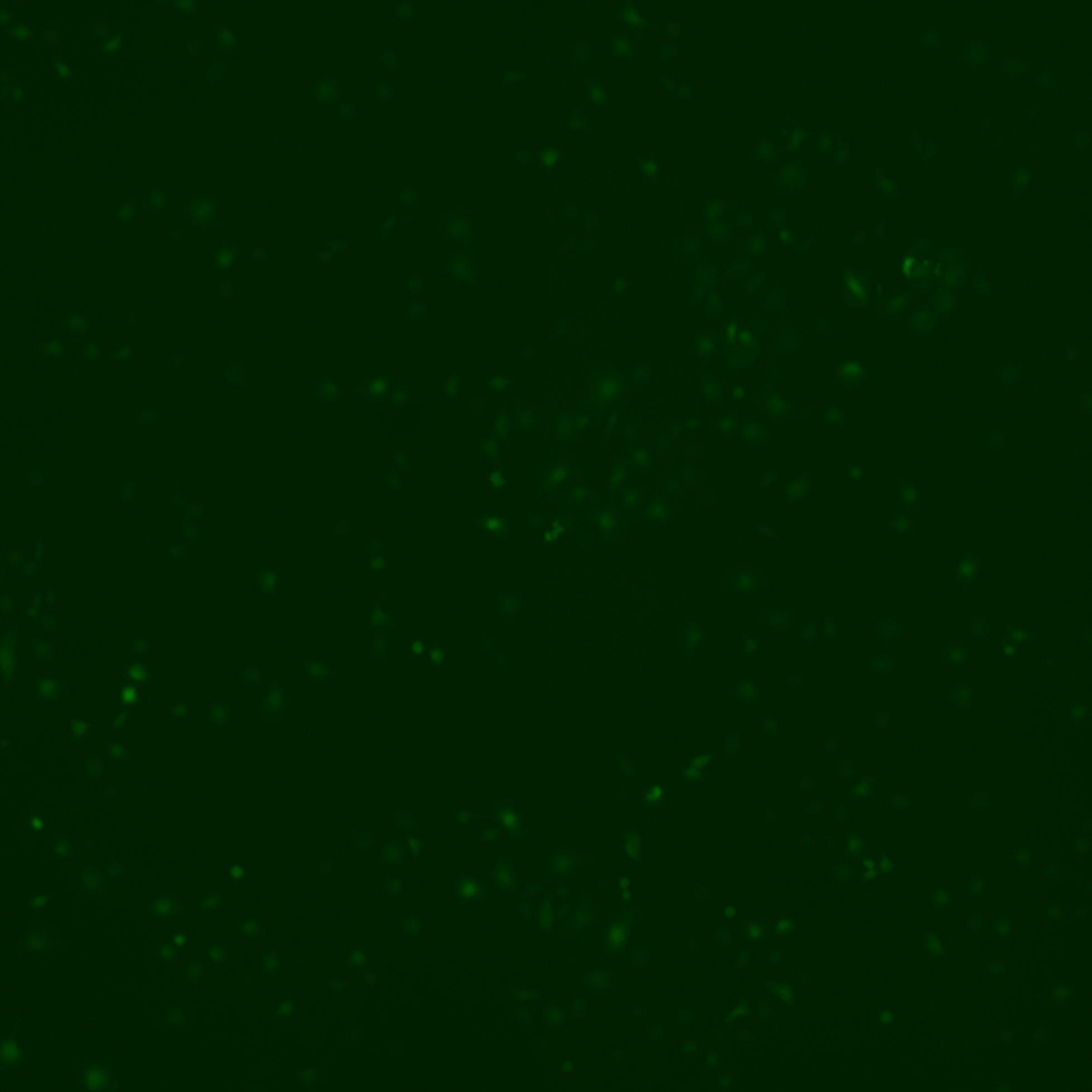

Supplement: Supplemental Information 12 [file peerj-10-12832-s012.zip › Original images 4 cell cultures/Figure1C-shCtrl-day2.jpg]

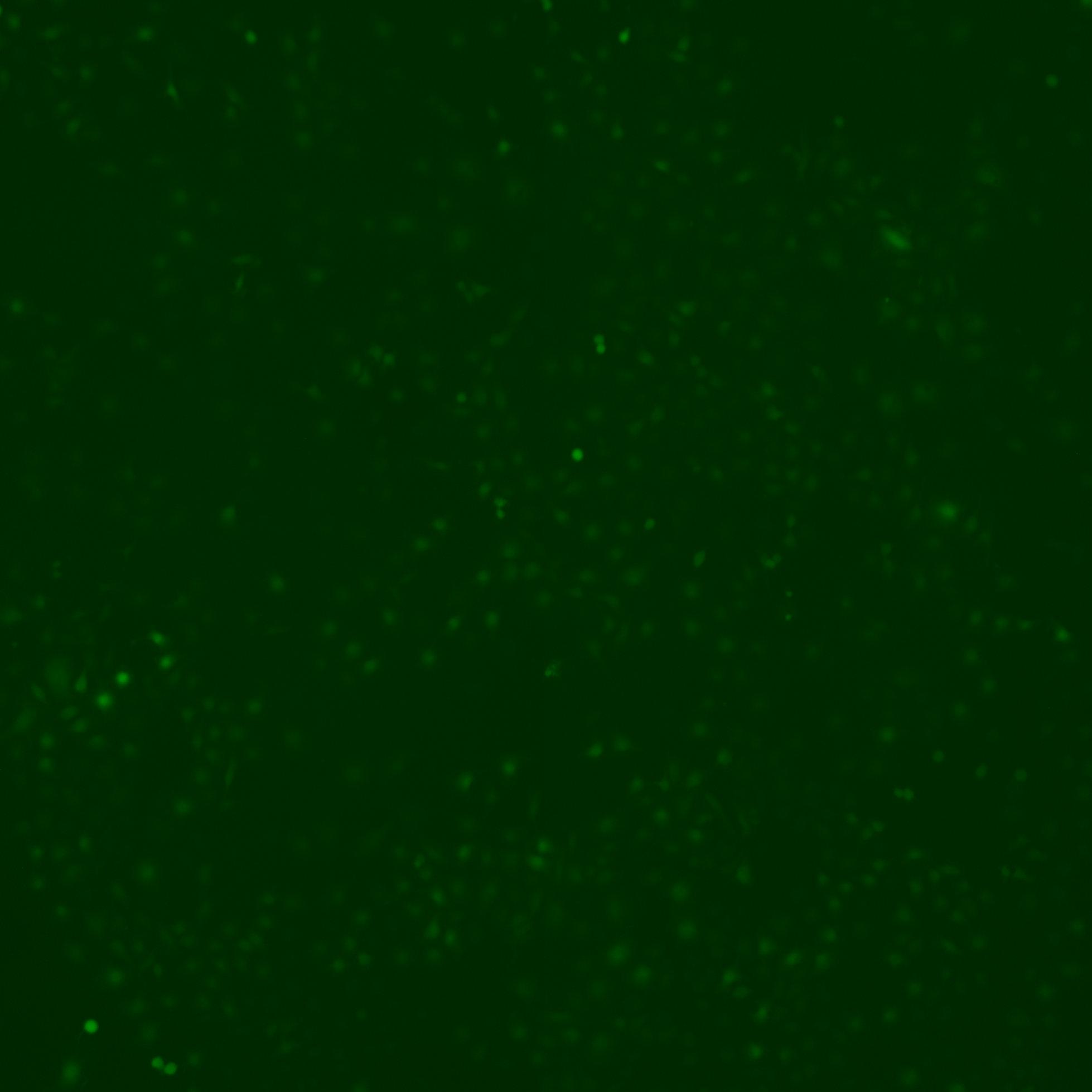

Supplement: Supplemental Information 12 [file peerj-10-12832-s012.zip › Original images 4 cell cultures/Figure1C-shCtrl-day3.jpg]

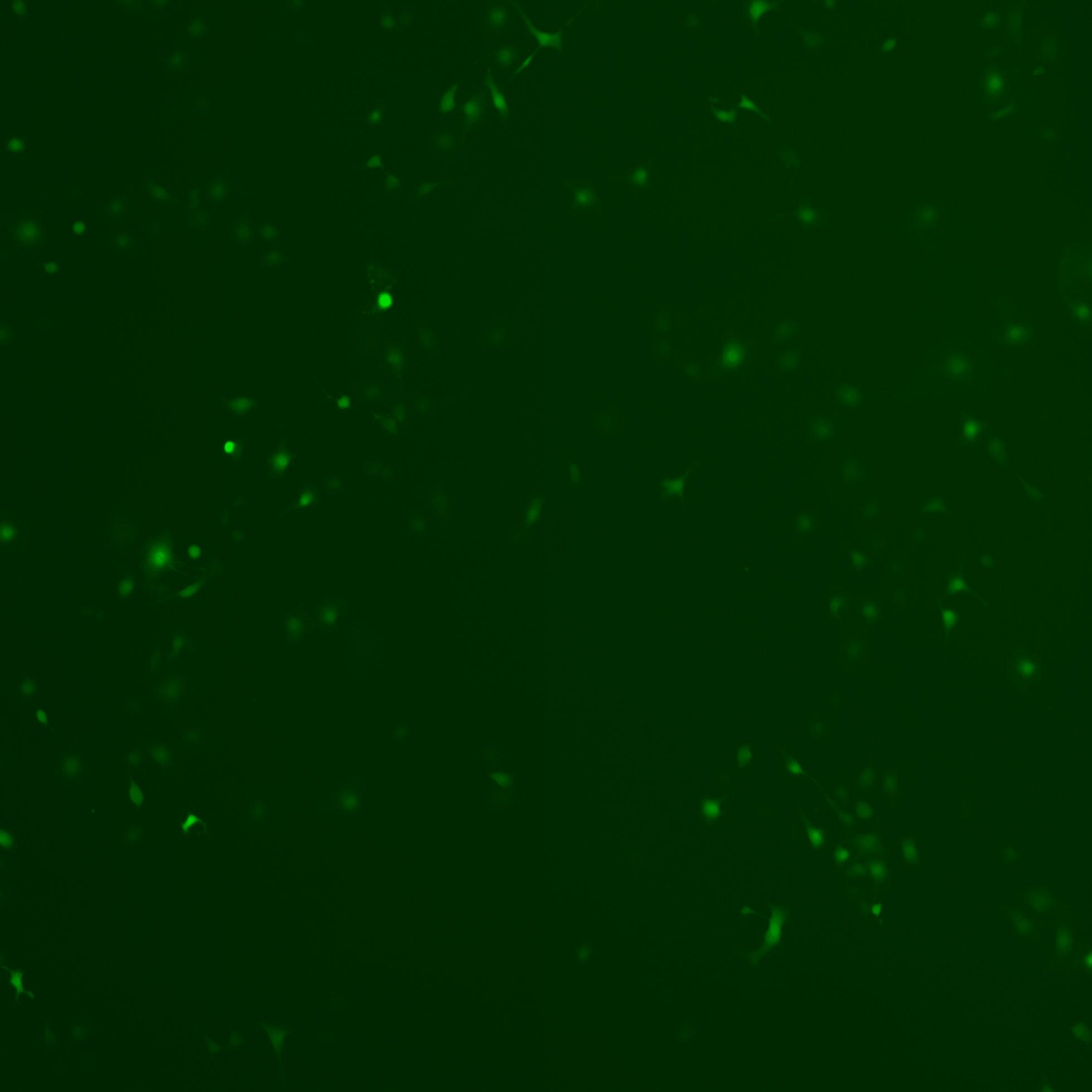

Supplement: Supplemental Information 12 [file peerj-10-12832-s012.zip › Original images 4 cell cultures/Figure1C-shDIDO1-day2.jpg]

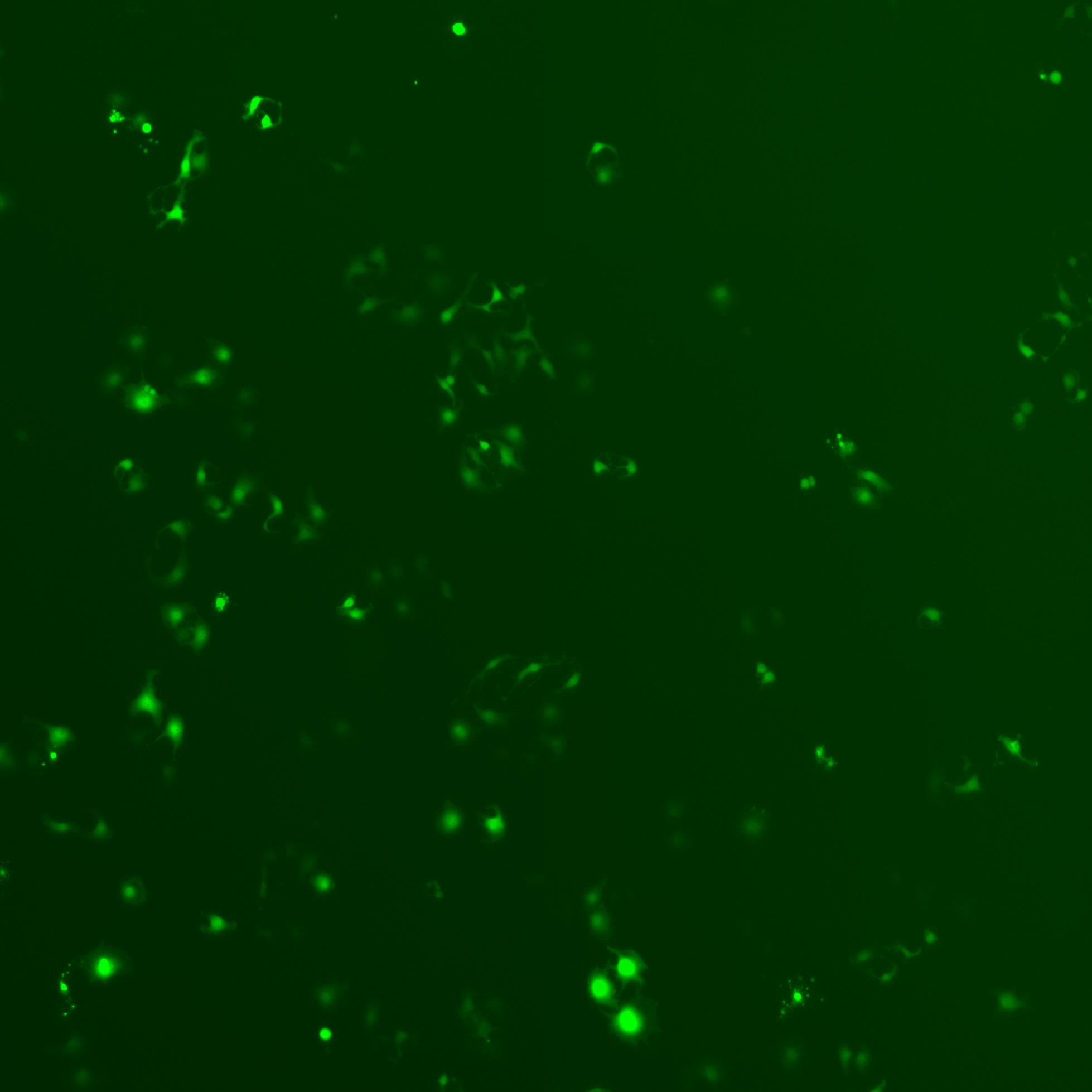

Supplement: Supplemental Information 12 [file peerj-10-12832-s012.zip › Original images 4 cell cultures/Figure S1A/FigureS1A-shZC3H18-day2.jpg]

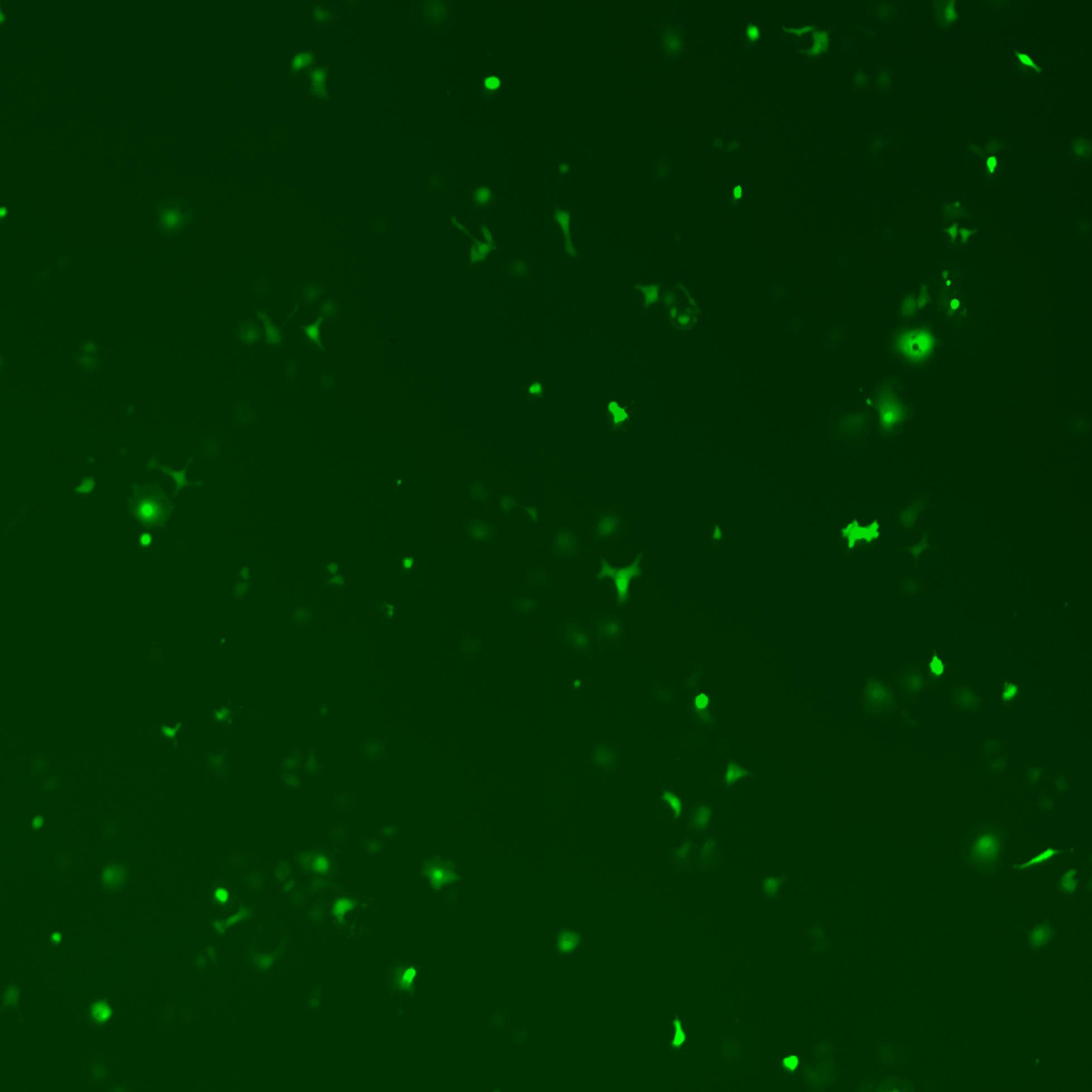

Supplement: Supplemental Information 12 [file peerj-10-12832-s012.zip › Original images 4 cell cultures/Figure S1A/FigureS1A-shPC-day1.jpg]

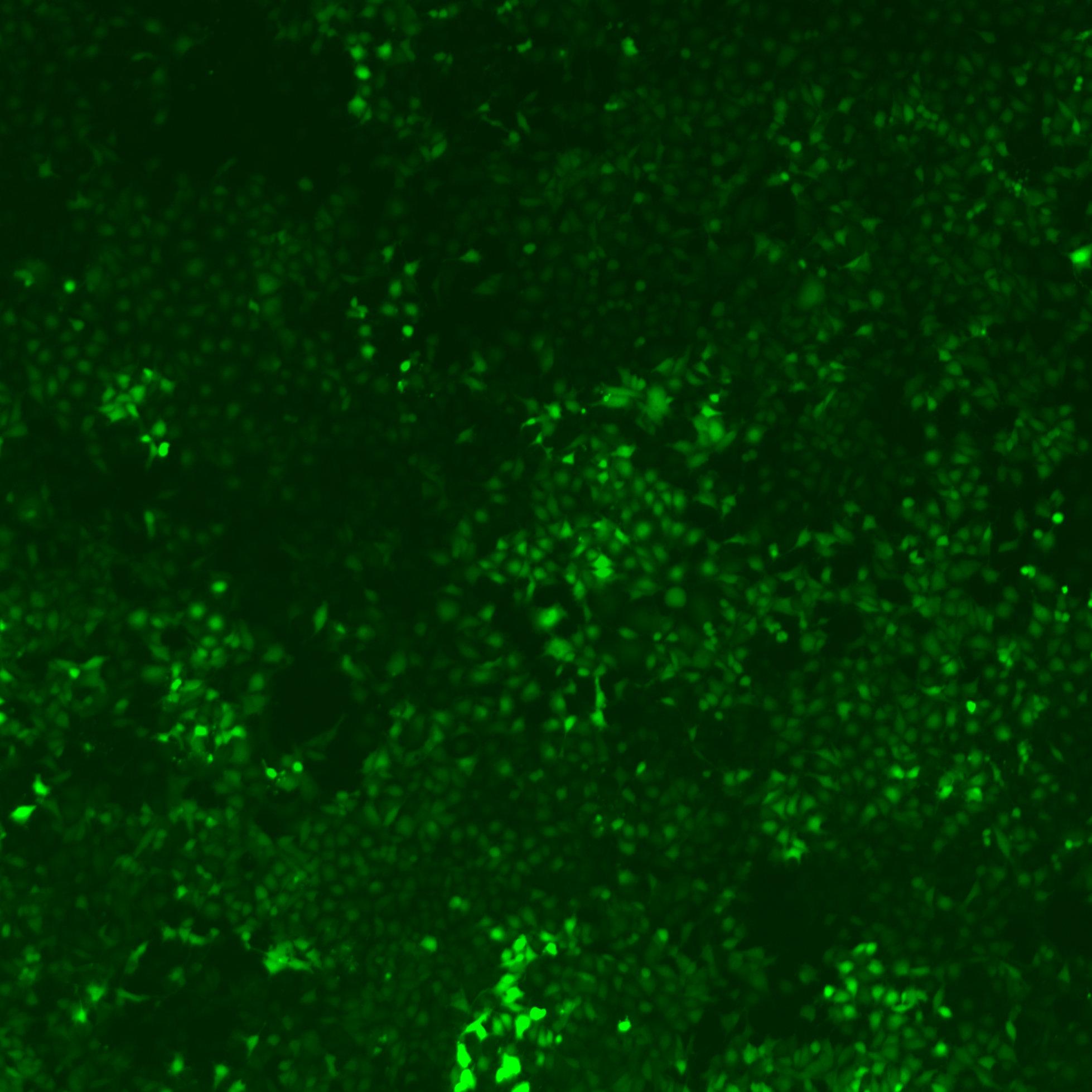

Supplement: Supplemental Information 12 [file peerj-10-12832-s012.zip › Original images 4 cell cultures/Figure S1A/FigureS1A-shCtrl-day5.jpg]

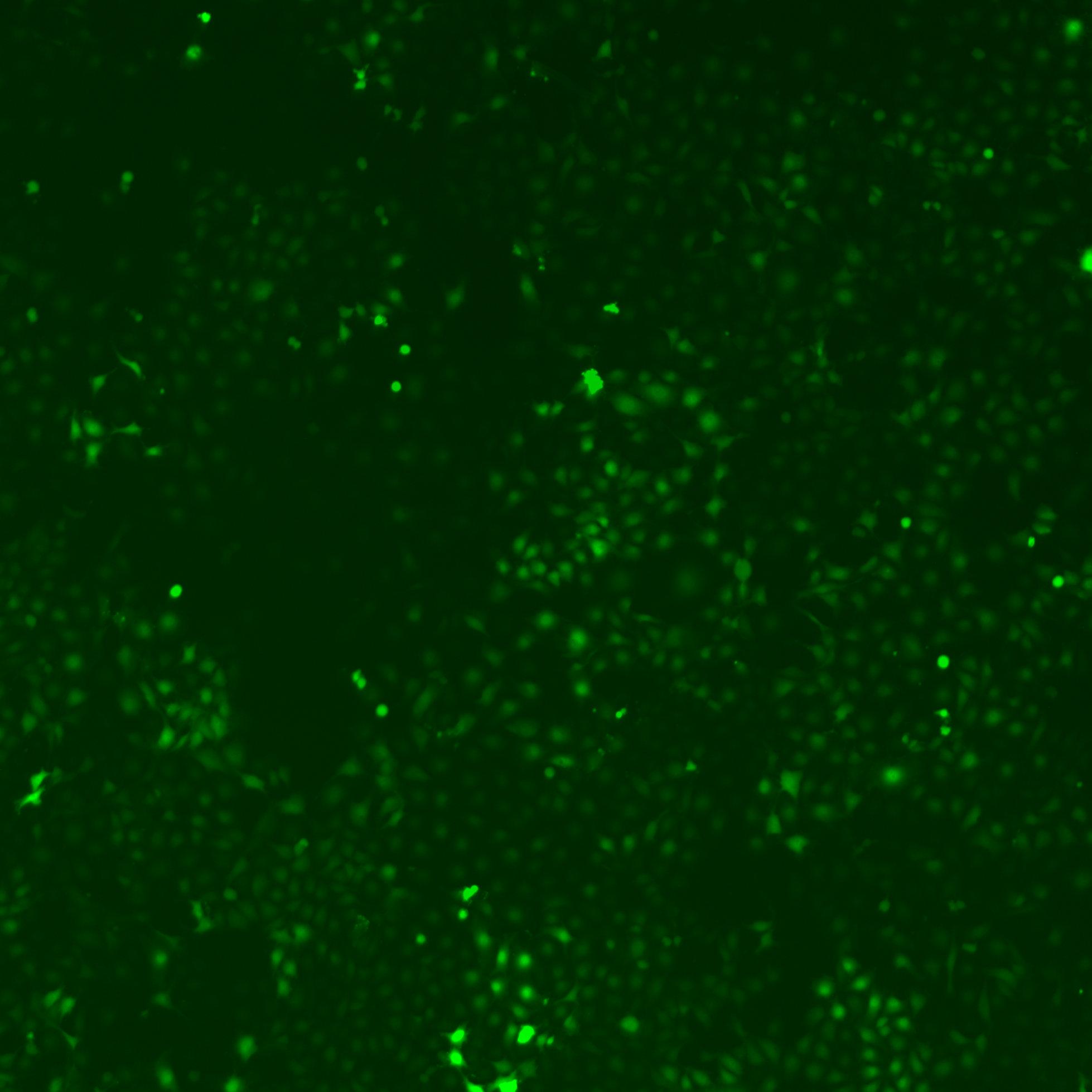

Supplement: Supplemental Information 12 [file peerj-10-12832-s012.zip › Original images 4 cell cultures/Figure S1A/FigureS1A-shCtrl-day4.jpg]

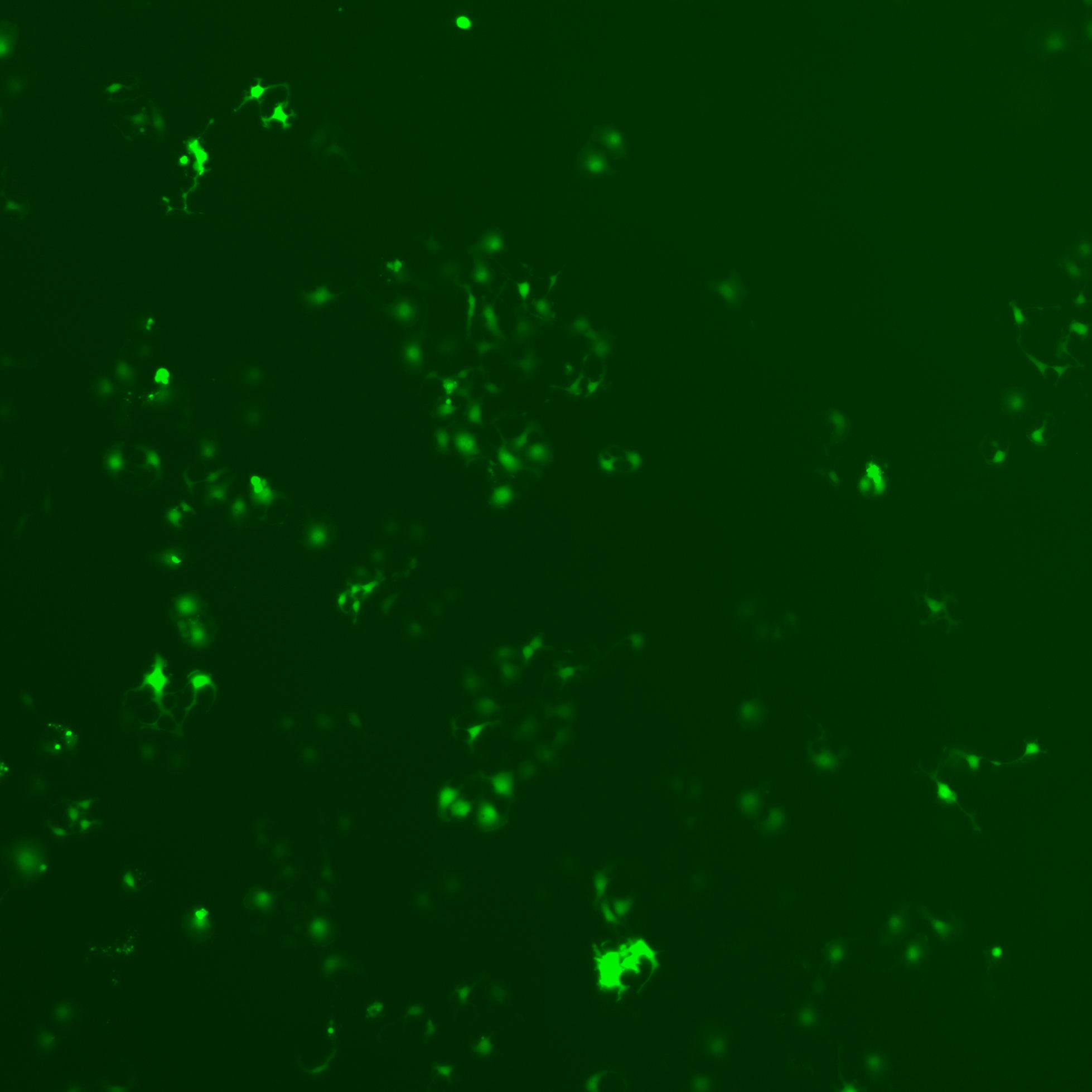

Supplement: Supplemental Information 12 [file peerj-10-12832-s012.zip › Original images 4 cell cultures/Figure S1A/FigureS1A-shZC3H18-day3.jpg]

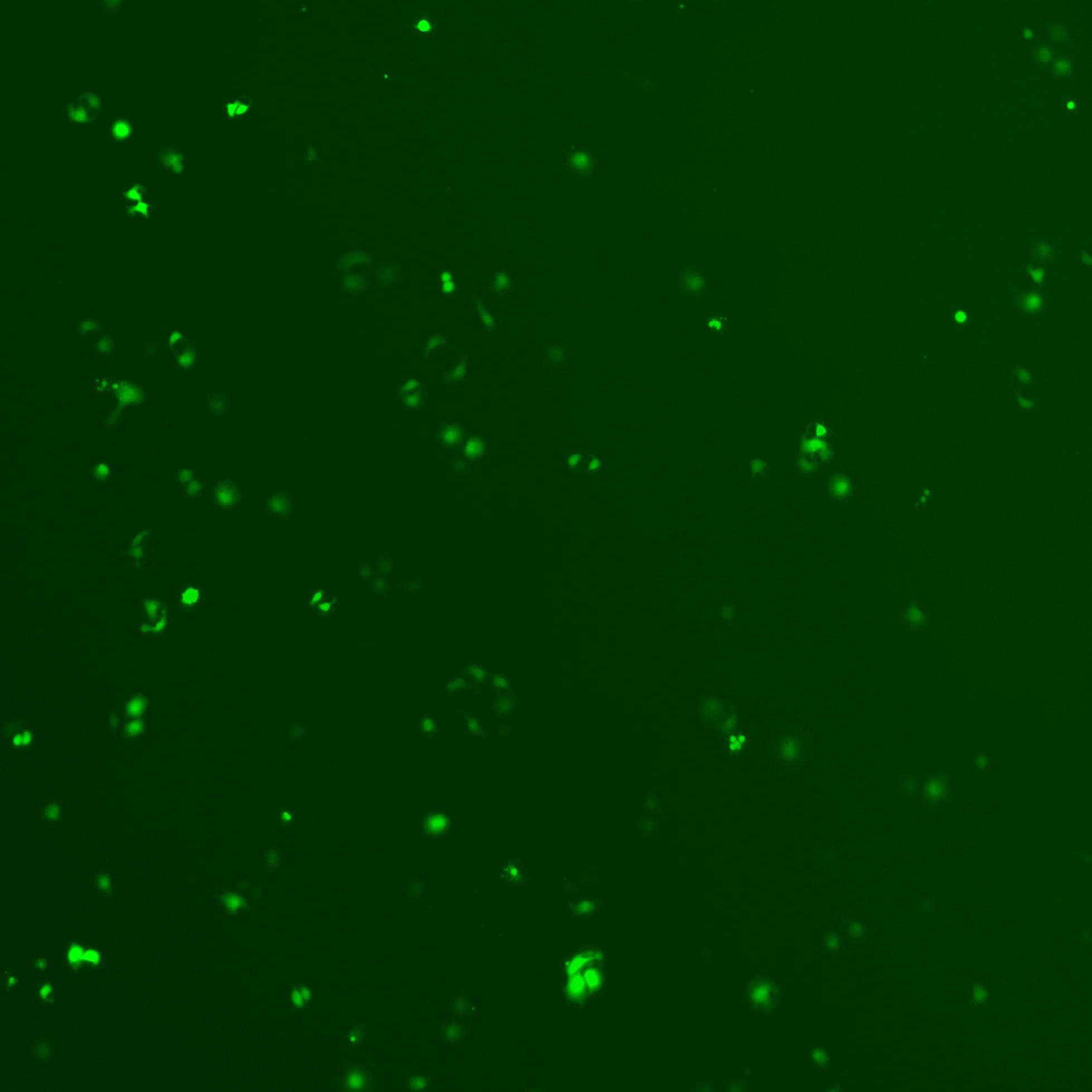

Supplement: Supplemental Information 12 [file peerj-10-12832-s012.zip › Original images 4 cell cultures/Figure S1A/FigureS1A-shZC3H18-day1.jpg]

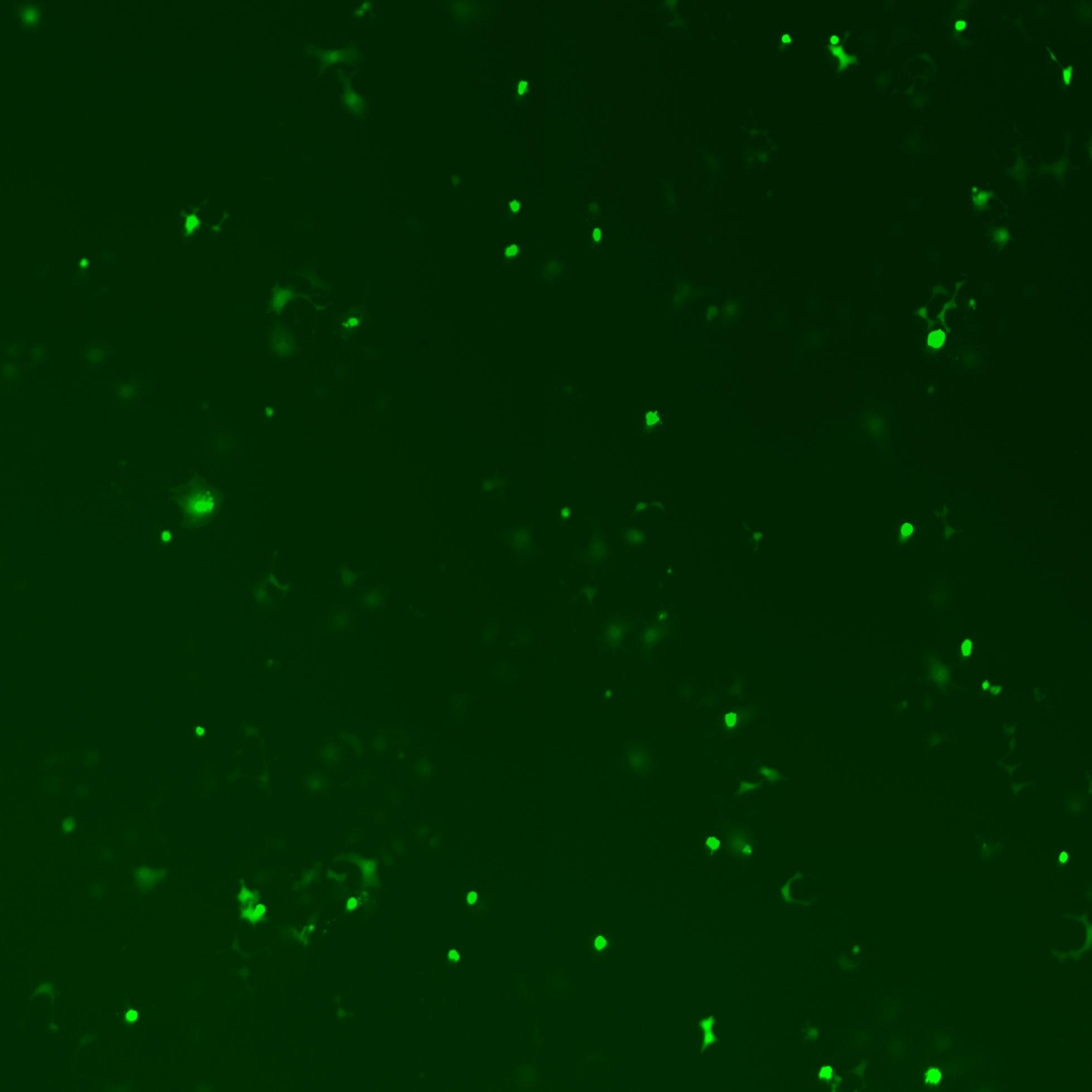

Supplement: Supplemental Information 12 [file peerj-10-12832-s012.zip › Original images 4 cell cultures/Figure S1A/FigureS1A-shPC-day2.jpg]

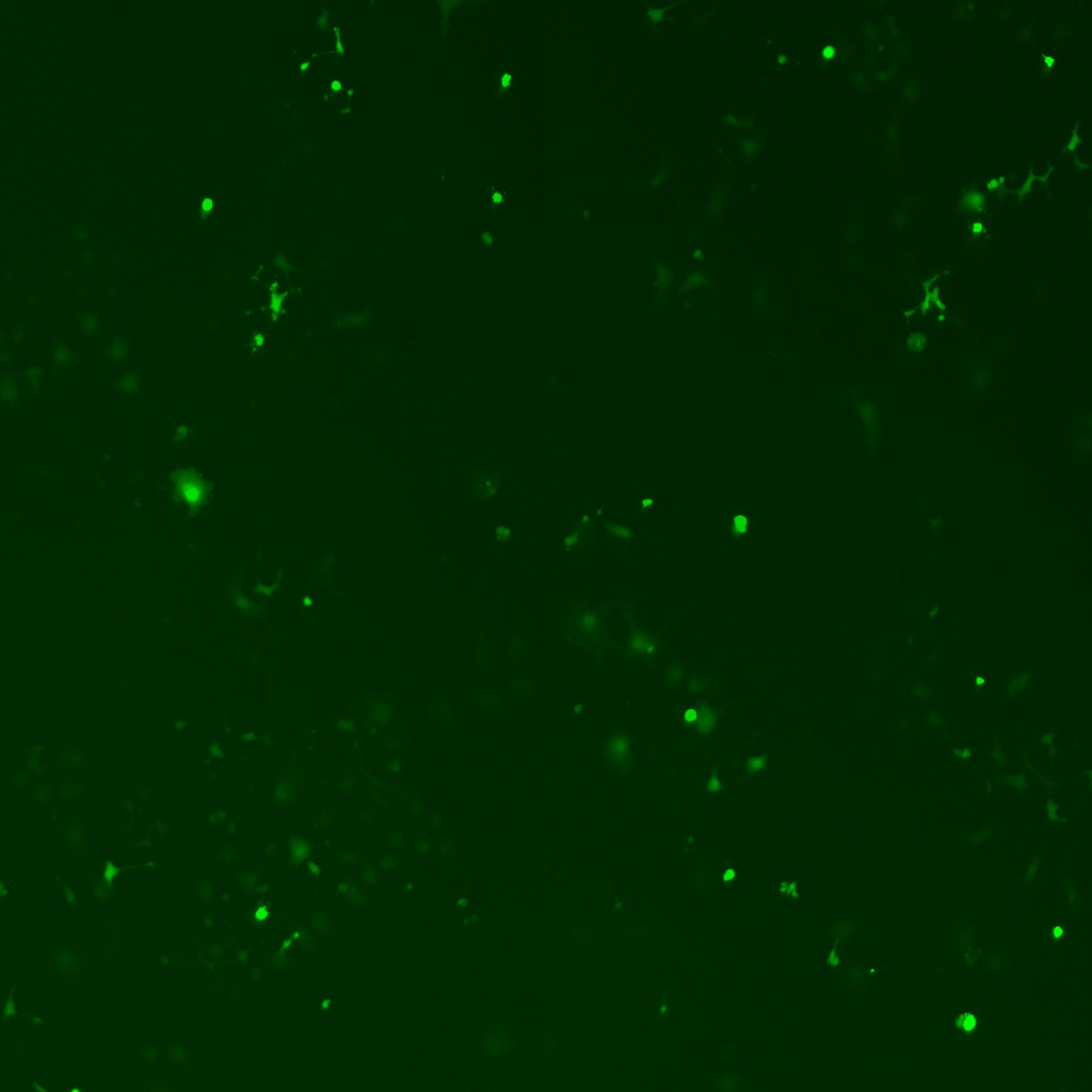

Supplement: Supplemental Information 12 [file peerj-10-12832-s012.zip › Original images 4 cell cultures/Figure S1A/FigureS1A-shPC-day3.jpg]

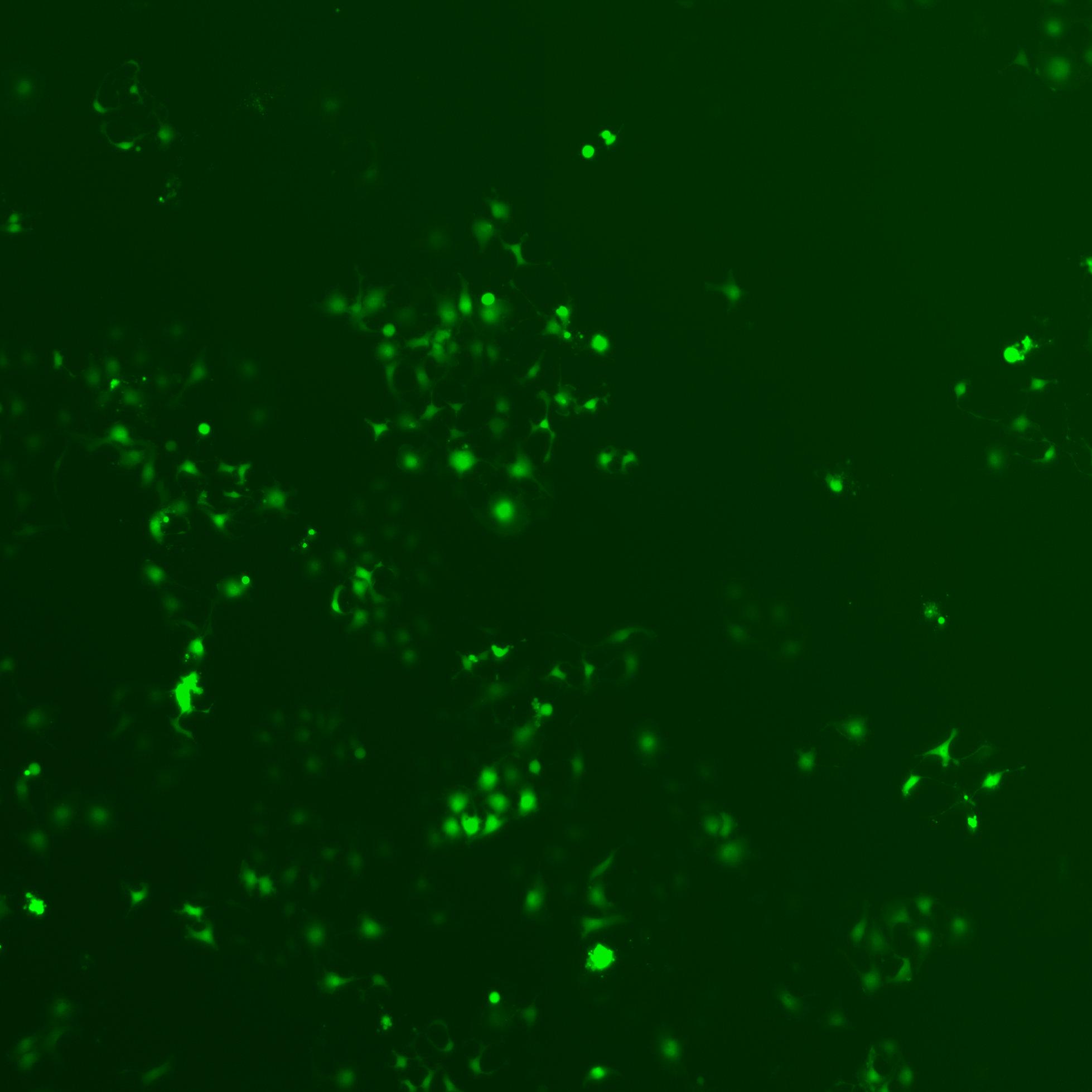

Supplement: Supplemental Information 12 [file peerj-10-12832-s012.zip › Original images 4 cell cultures/Figure S1A/FigureS1A-shZC3H18-day4.jpg]

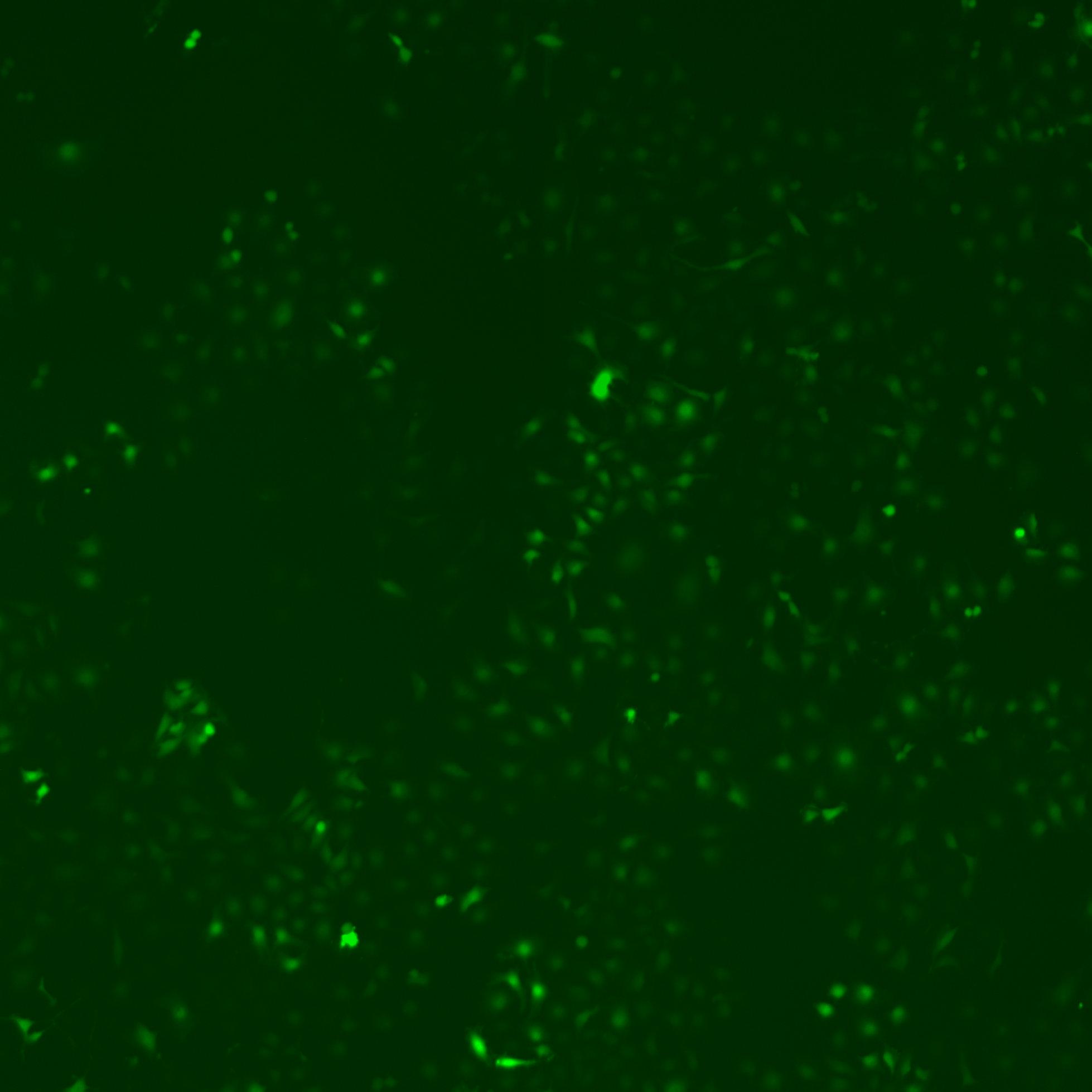

Supplement: Supplemental Information 12 [file peerj-10-12832-s012.zip › Original images 4 cell cultures/Figure S1A/FigureS1A-shCtrl-day3.jpg]

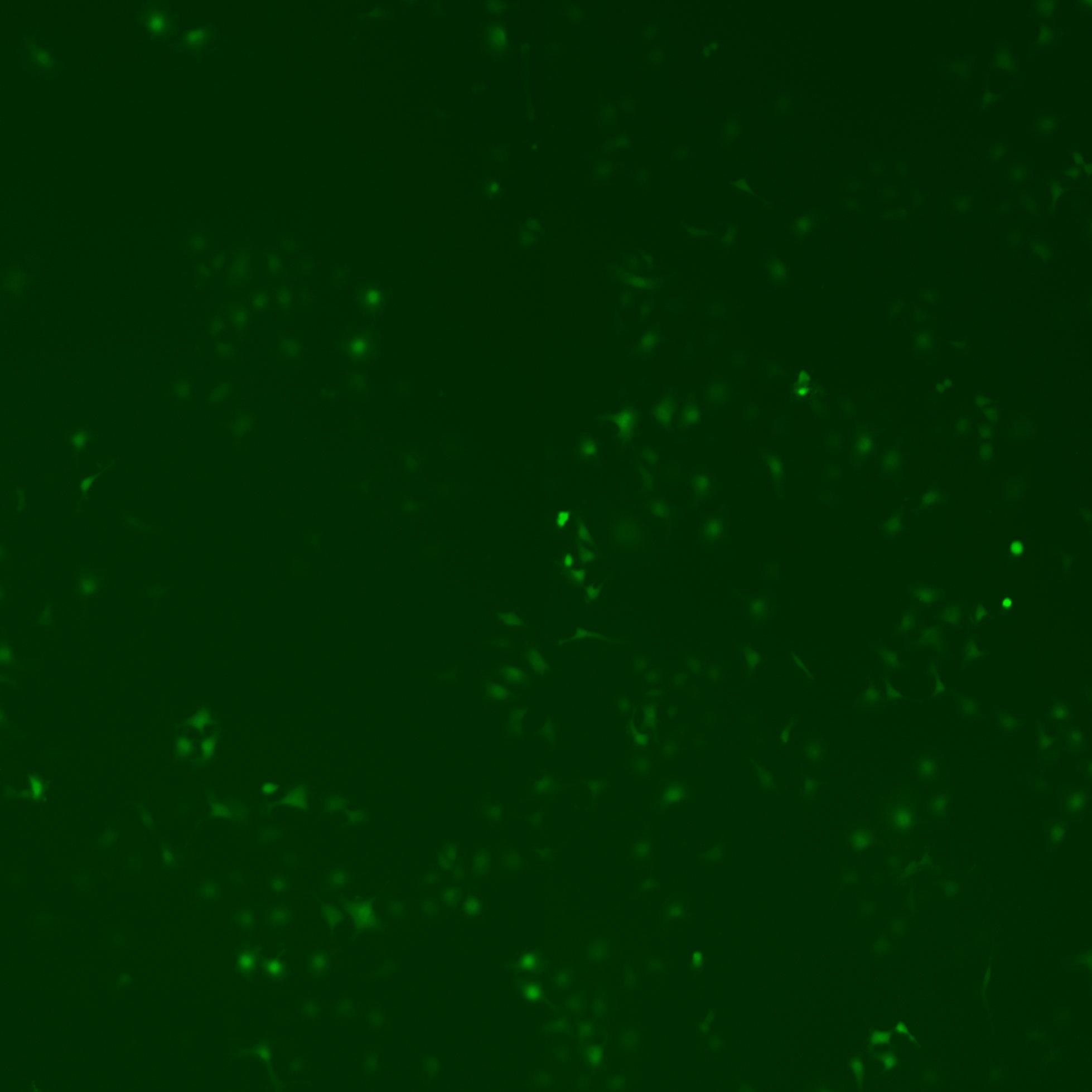

Supplement: Supplemental Information 12 [file peerj-10-12832-s012.zip › Original images 4 cell cultures/Figure S1A/FigureS1A-shCtrl-day2.jpg]

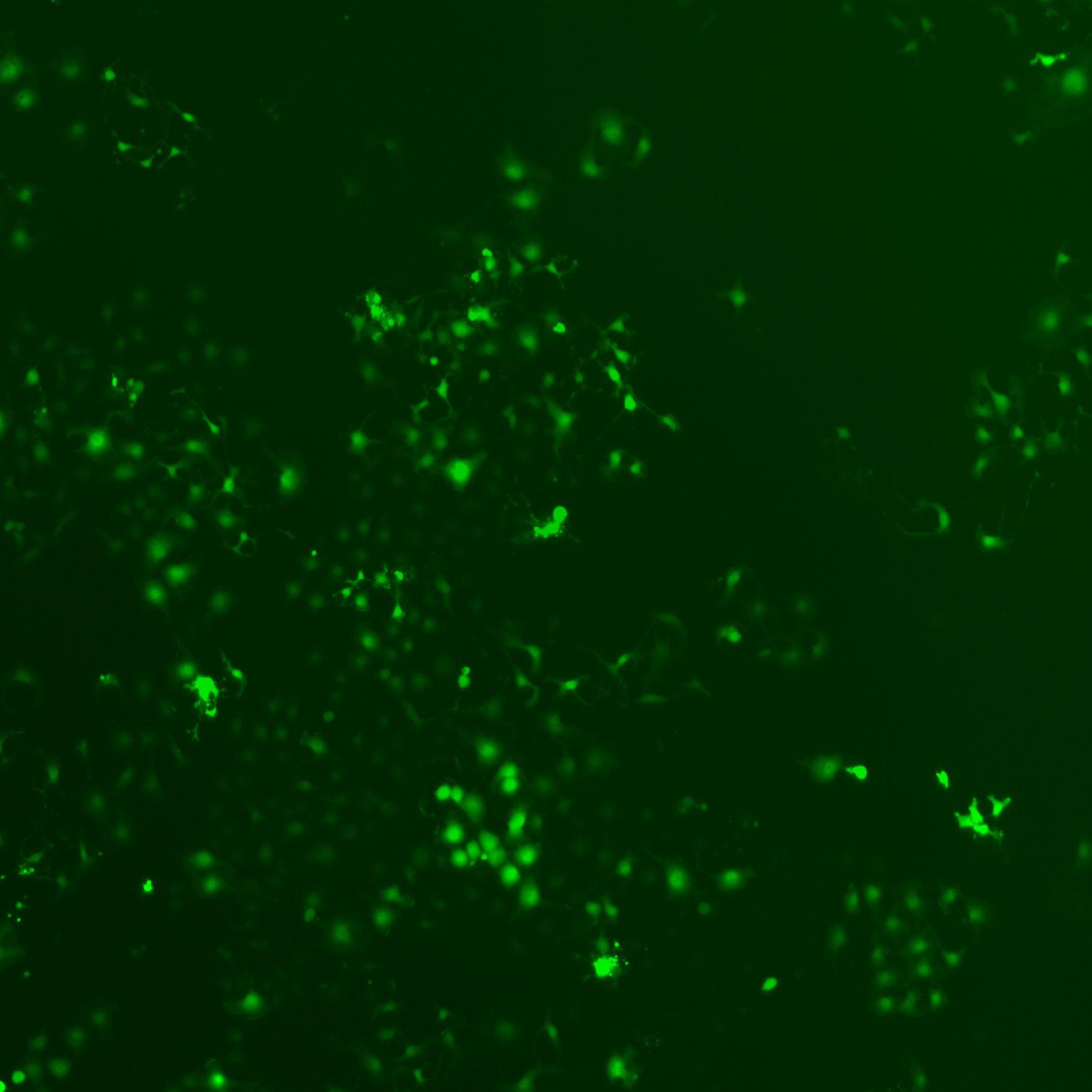

Supplement: Supplemental Information 12 [file peerj-10-12832-s012.zip › Original images 4 cell cultures/Figure S1A/FigureS1A-shZC3H18-day5.jpg]

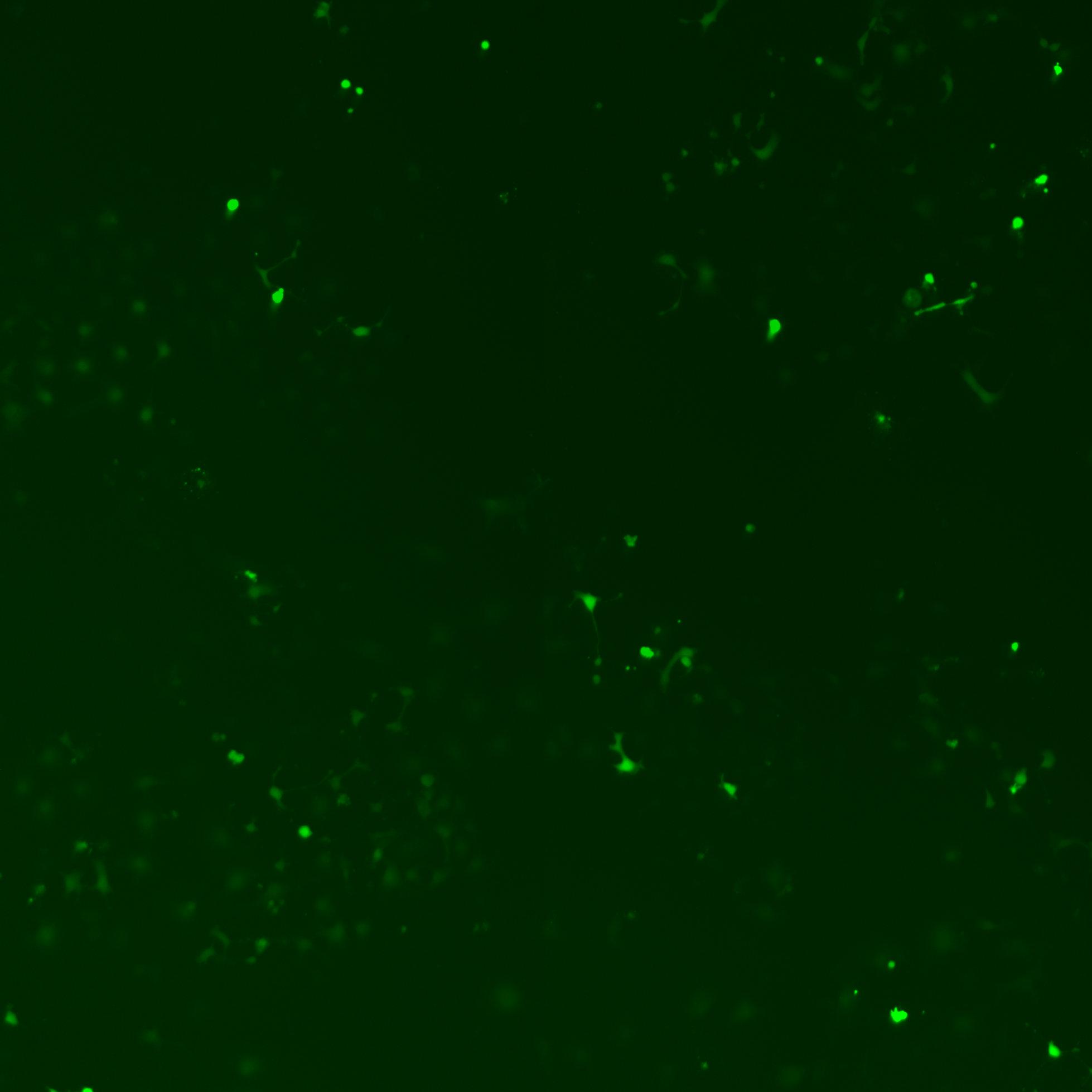

Supplement: Supplemental Information 12 [file peerj-10-12832-s012.zip › Original images 4 cell cultures/Figure S1A/FigureS1A-shPC-day4.jpg]

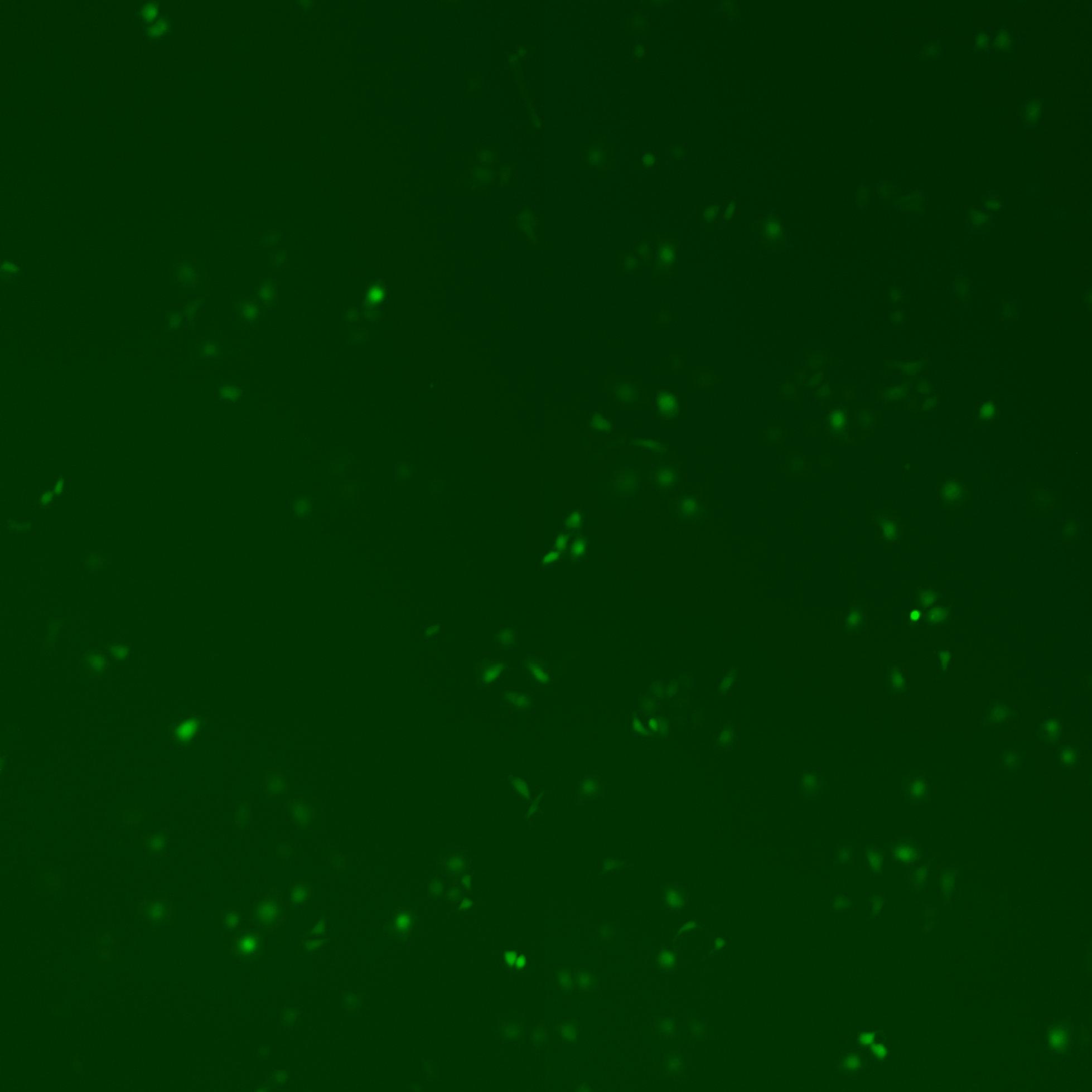

Supplement: Supplemental Information 12 [file peerj-10-12832-s012.zip › Original images 4 cell cultures/Figure S1A/FigureS1A-shCtrl-day1.jpg]

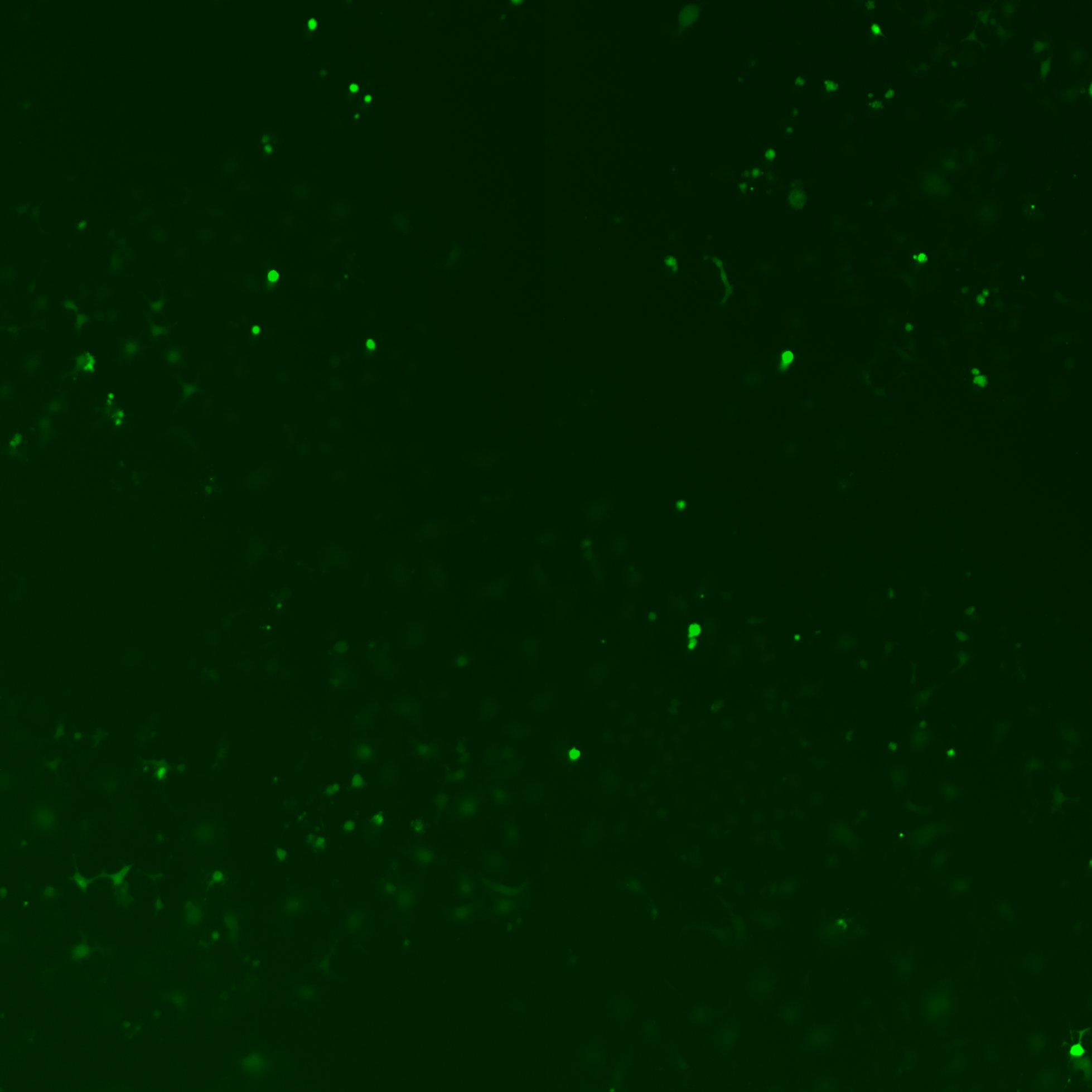

Supplement: Supplemental Information 12 [file peerj-10-12832-s012.zip › Original images 4 cell cultures/Figure S1A/FigureS1A-shPC-day5.jpg]

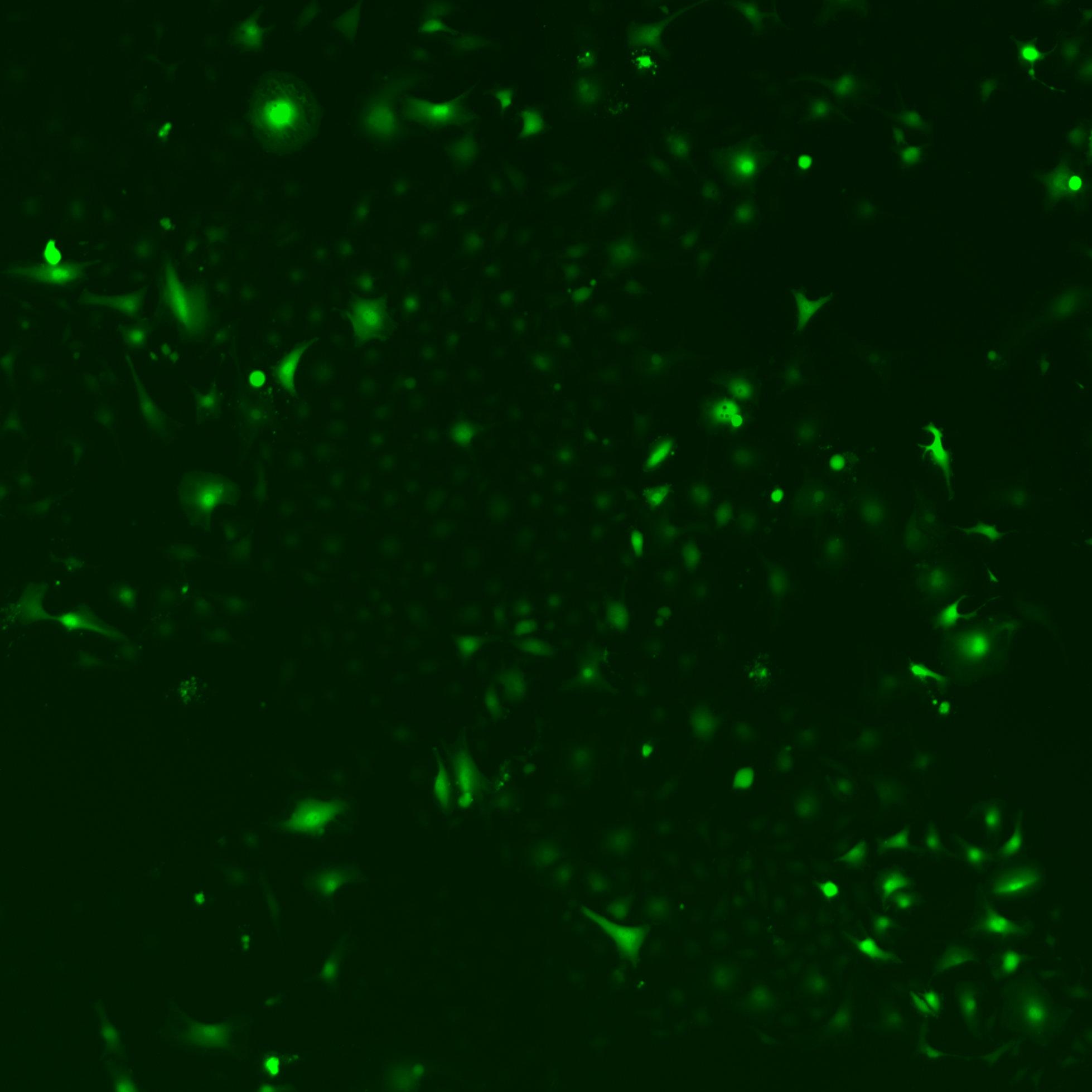

Supplement: Supplemental Information 12 [file peerj-10-12832-s012.zip › Original images 4 cell cultures/Figure S1A/FigureS1A-shSMURF2-day5.jpg]

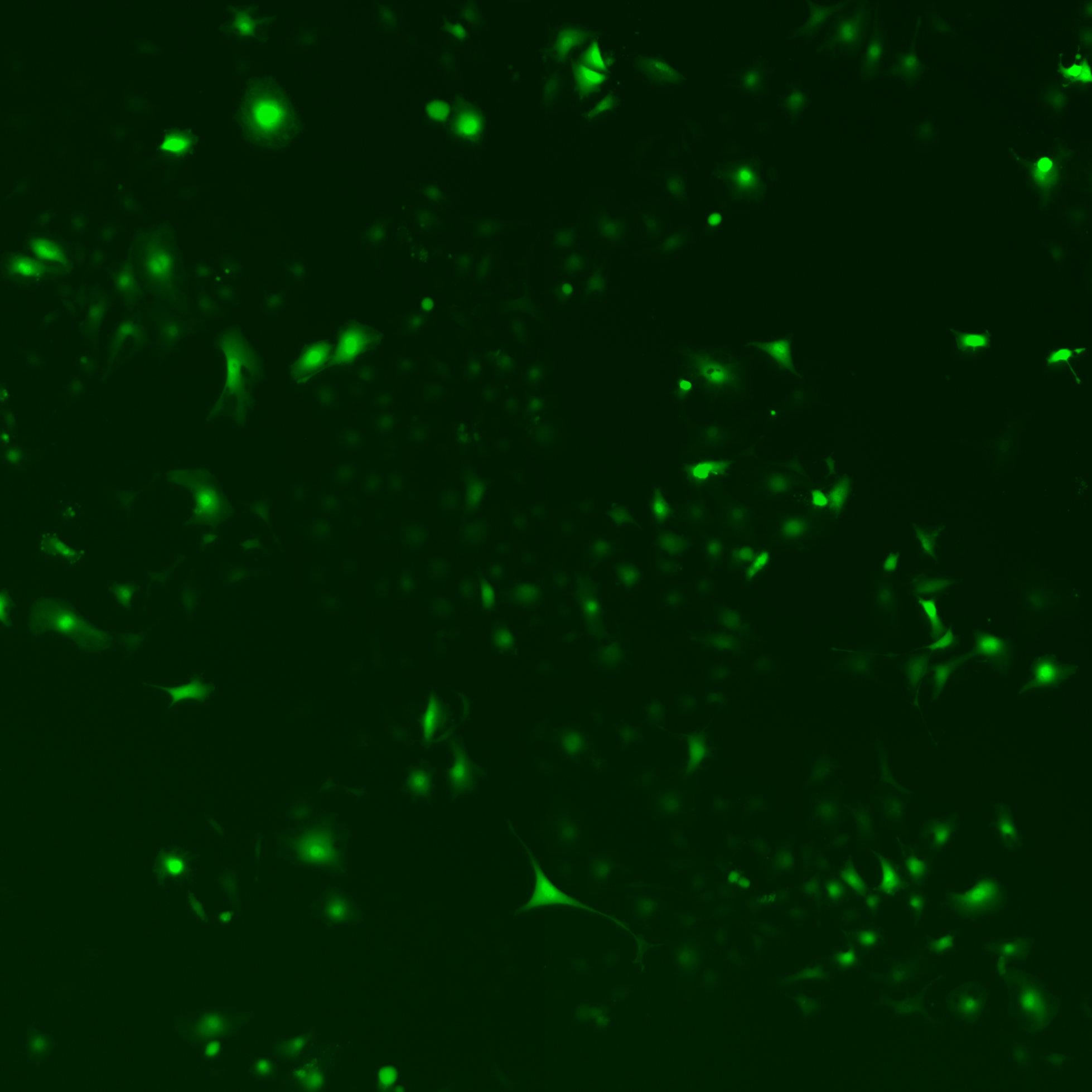

Supplement: Supplemental Information 12 [file peerj-10-12832-s012.zip › Original images 4 cell cultures/Figure S1A/FigureS1A-shSMURF2-day4.jpg]

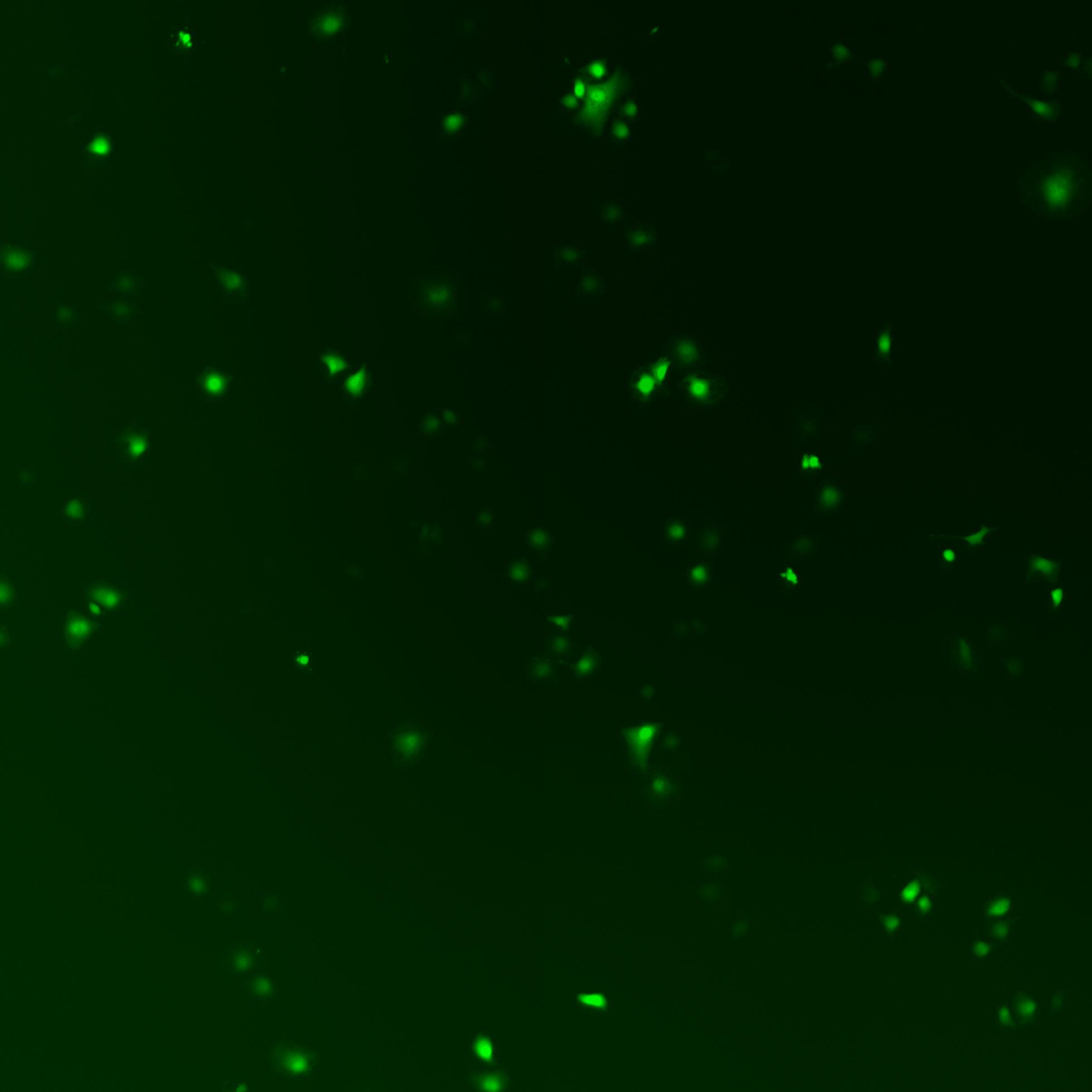

Supplement: Supplemental Information 12 [file peerj-10-12832-s012.zip › Original images 4 cell cultures/Figure S1A/FigureS1A-shSMURF2-day1.jpg]

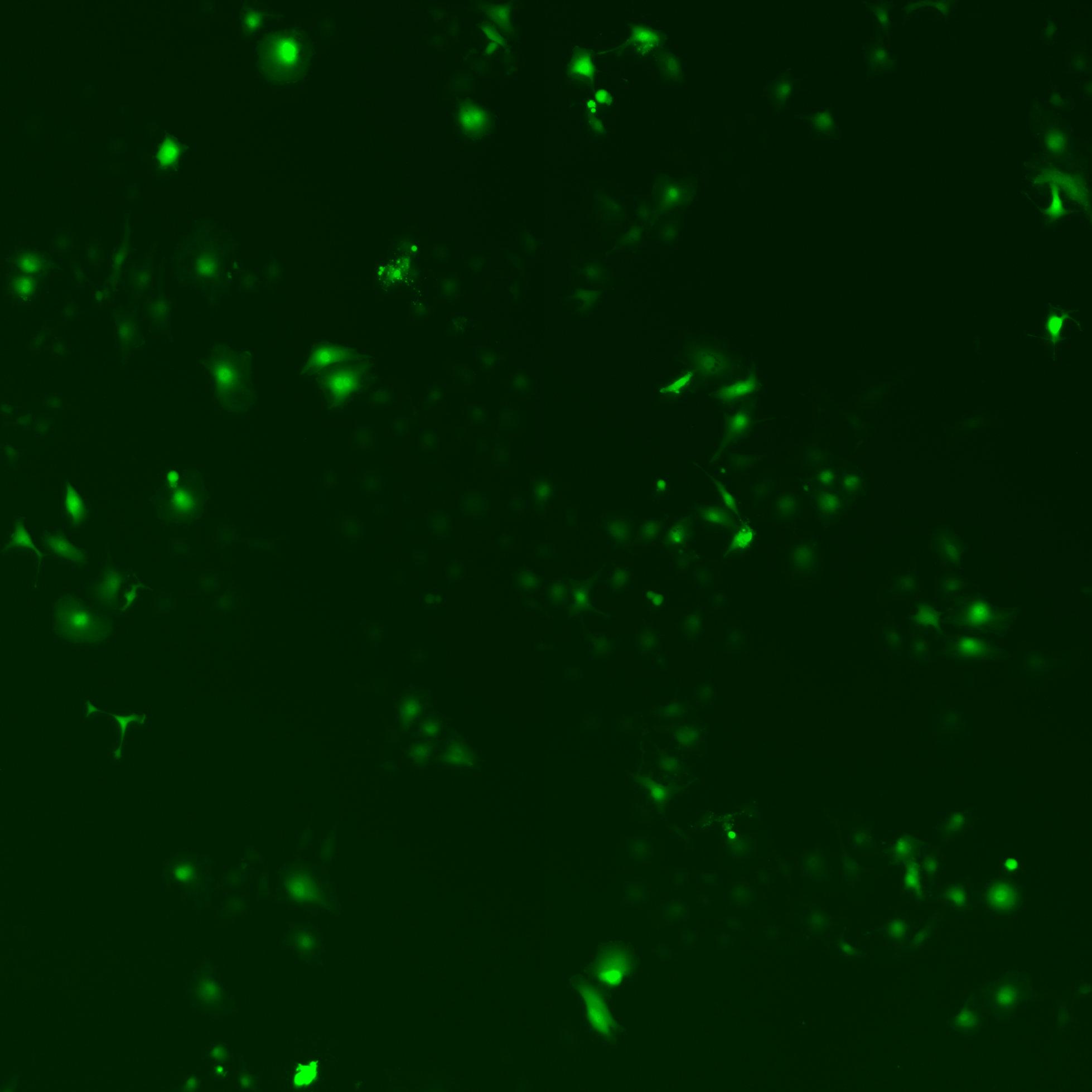

Supplement: Supplemental Information 12 [file peerj-10-12832-s012.zip › Original images 4 cell cultures/Figure S1A/FigureS1A-shSMURF2-day3.jpg]

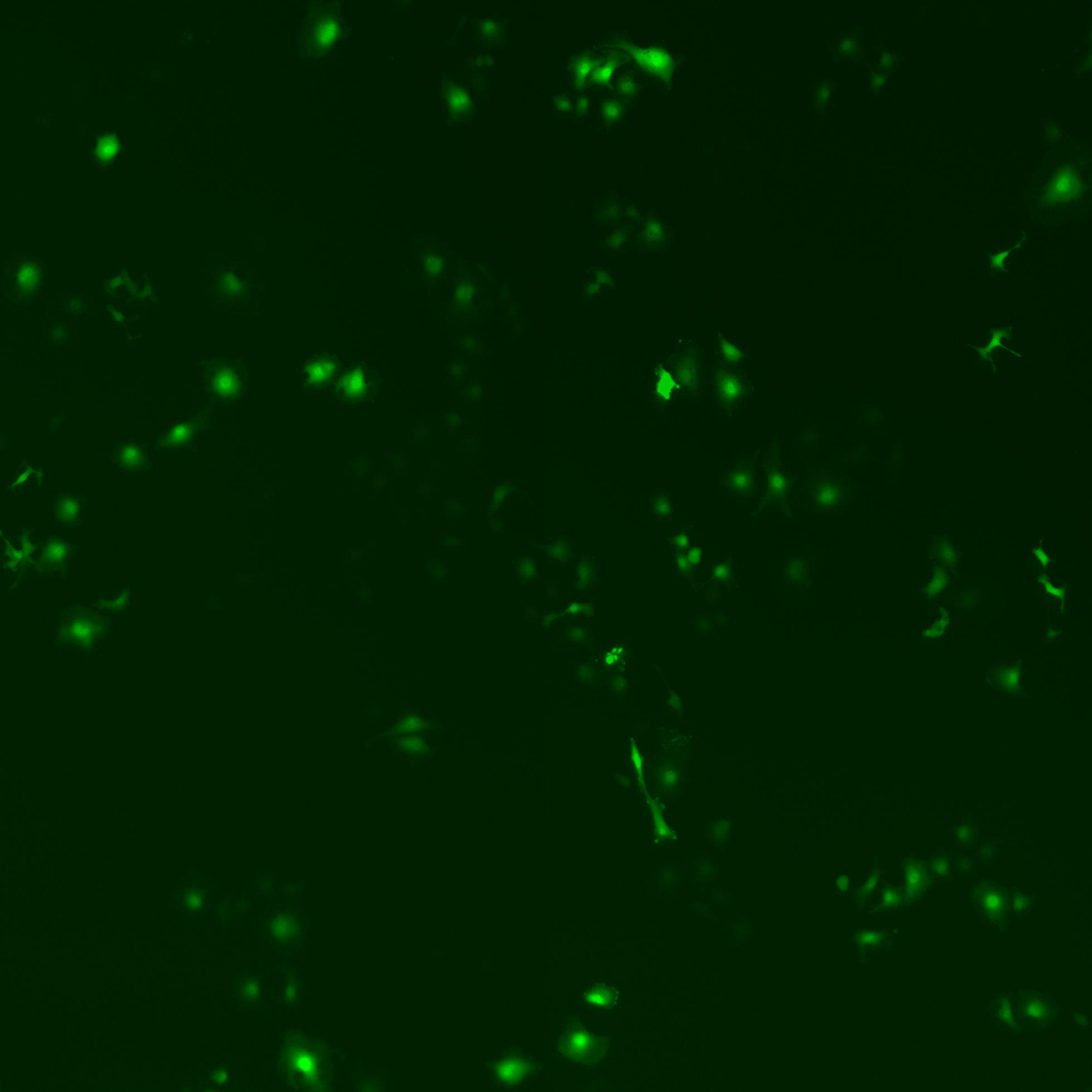

Supplement: Supplemental Information 12 [file peerj-10-12832-s012.zip › Original images 4 cell cultures/Figure S1A/FigureS1A-shSMURF2-day2.jpg]

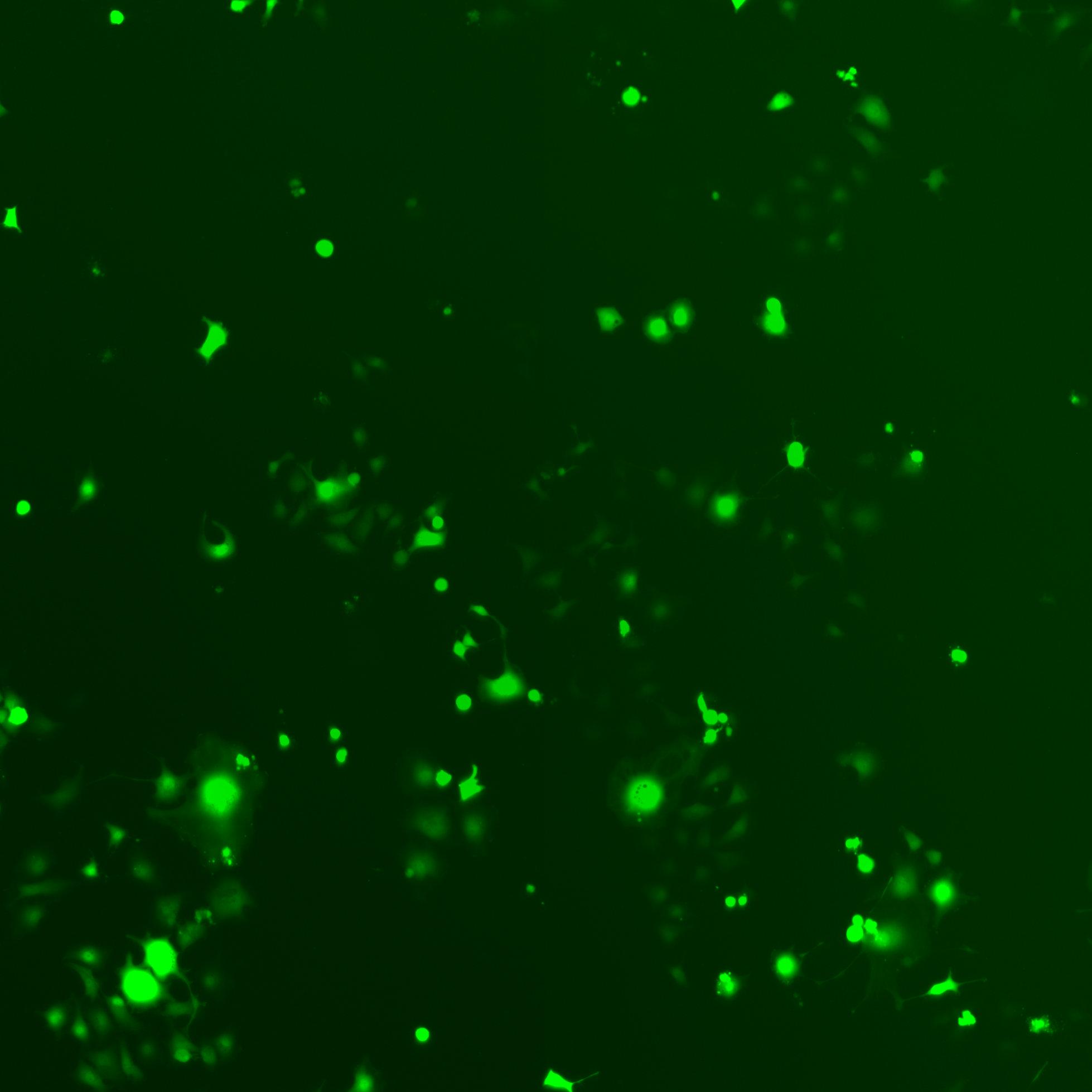

Supplement: Supplemental Information 12 [file peerj-10-12832-s012.zip › Original images 4 cell cultures/Figure S1A/FigureS1A-shDIDO1-day3.jpg]

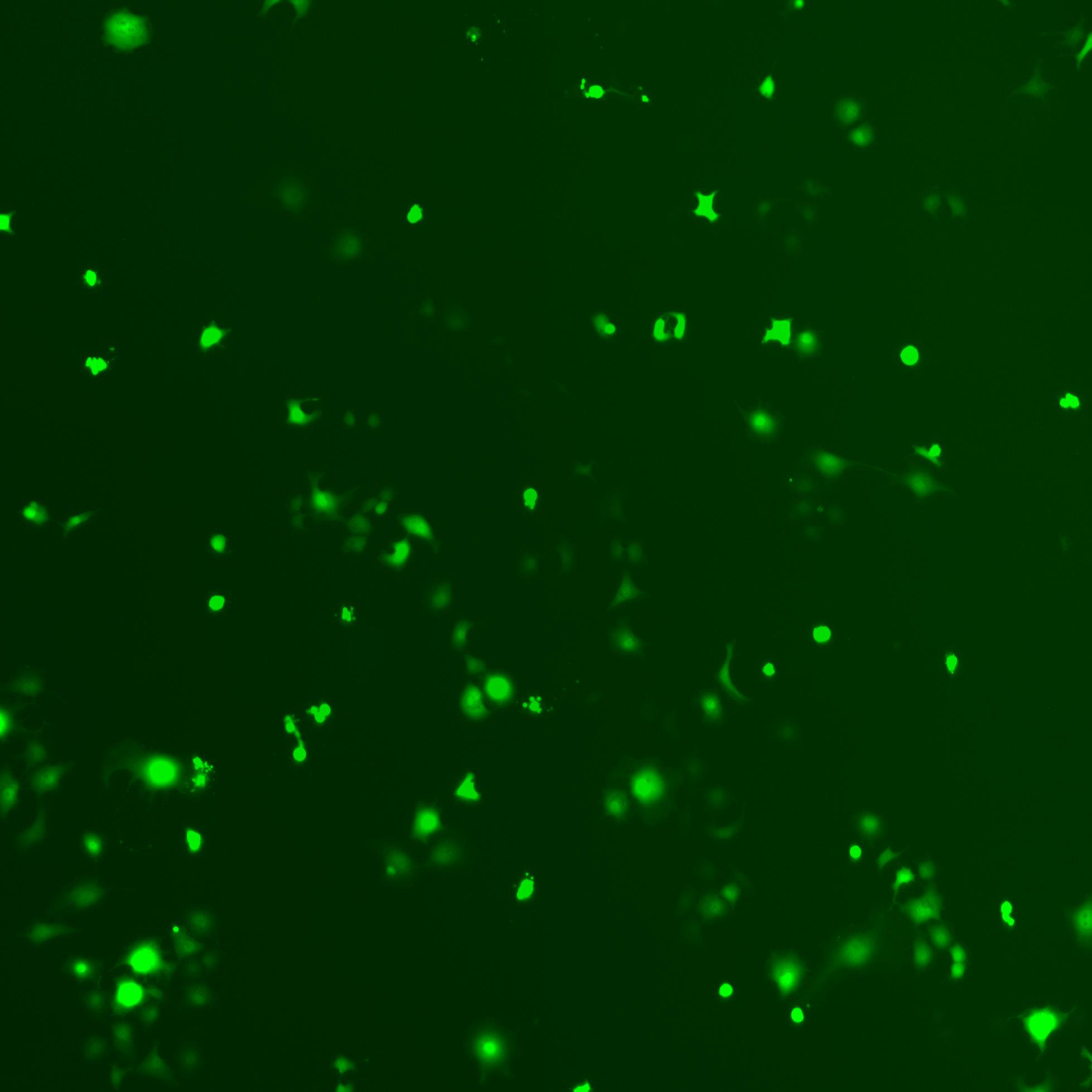

Supplement: Supplemental Information 12 [file peerj-10-12832-s012.zip › Original images 4 cell cultures/Figure S1A/FigureS1A-shDIDO1-day2.jpg]

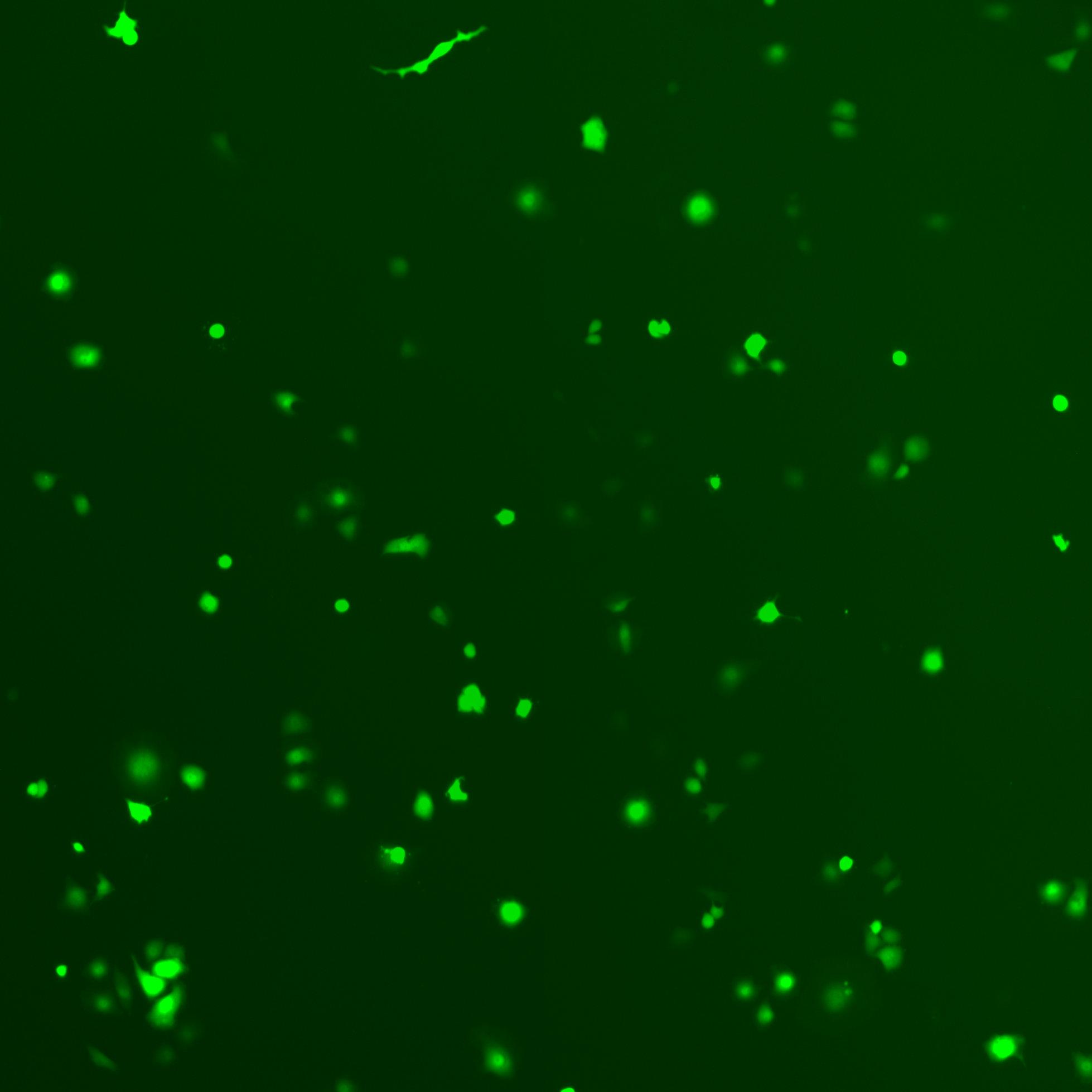

Supplement: Supplemental Information 12 [file peerj-10-12832-s012.zip › Original images 4 cell cultures/Figure S1A/FigureS1A-shDIDO1-day1.jpg]

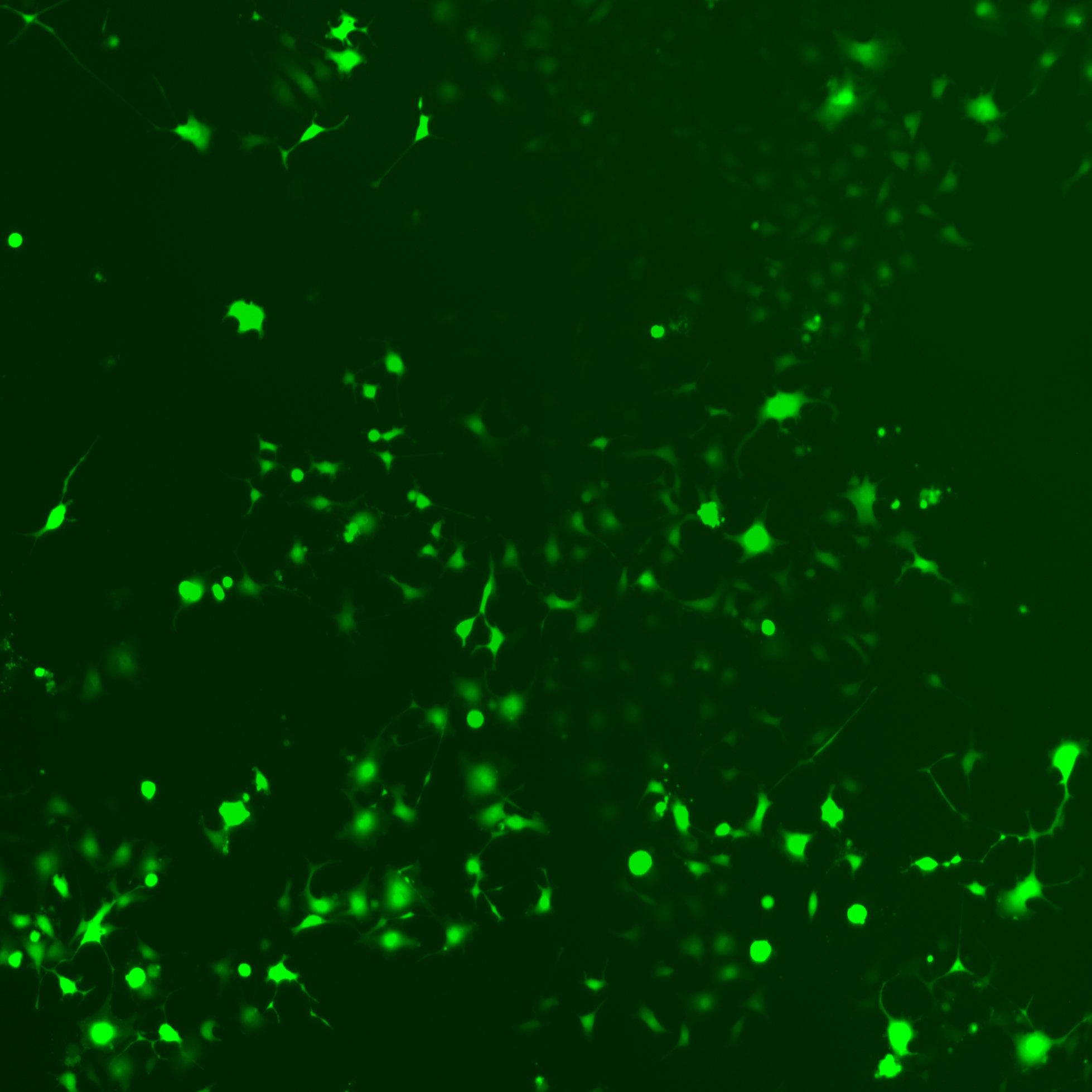

Supplement: Supplemental Information 12 [file peerj-10-12832-s012.zip › Original images 4 cell cultures/Figure S1A/FigureS1A-shDIDO1-day5.jpg]

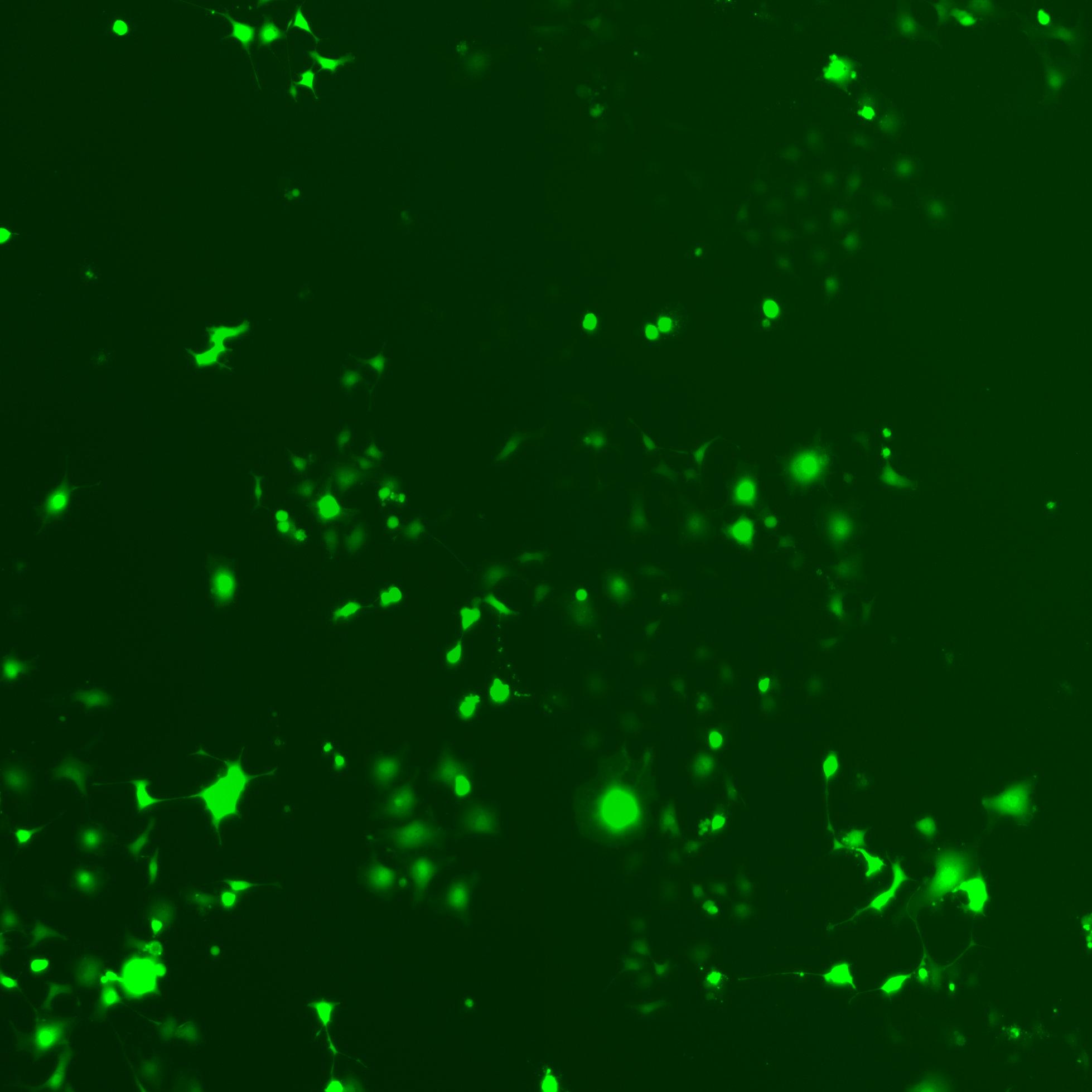

Supplement: Supplemental Information 12 [file peerj-10-12832-s012.zip › Original images 4 cell cultures/Figure S1A/FigureS1A-shDIDO1-day4.jpg]

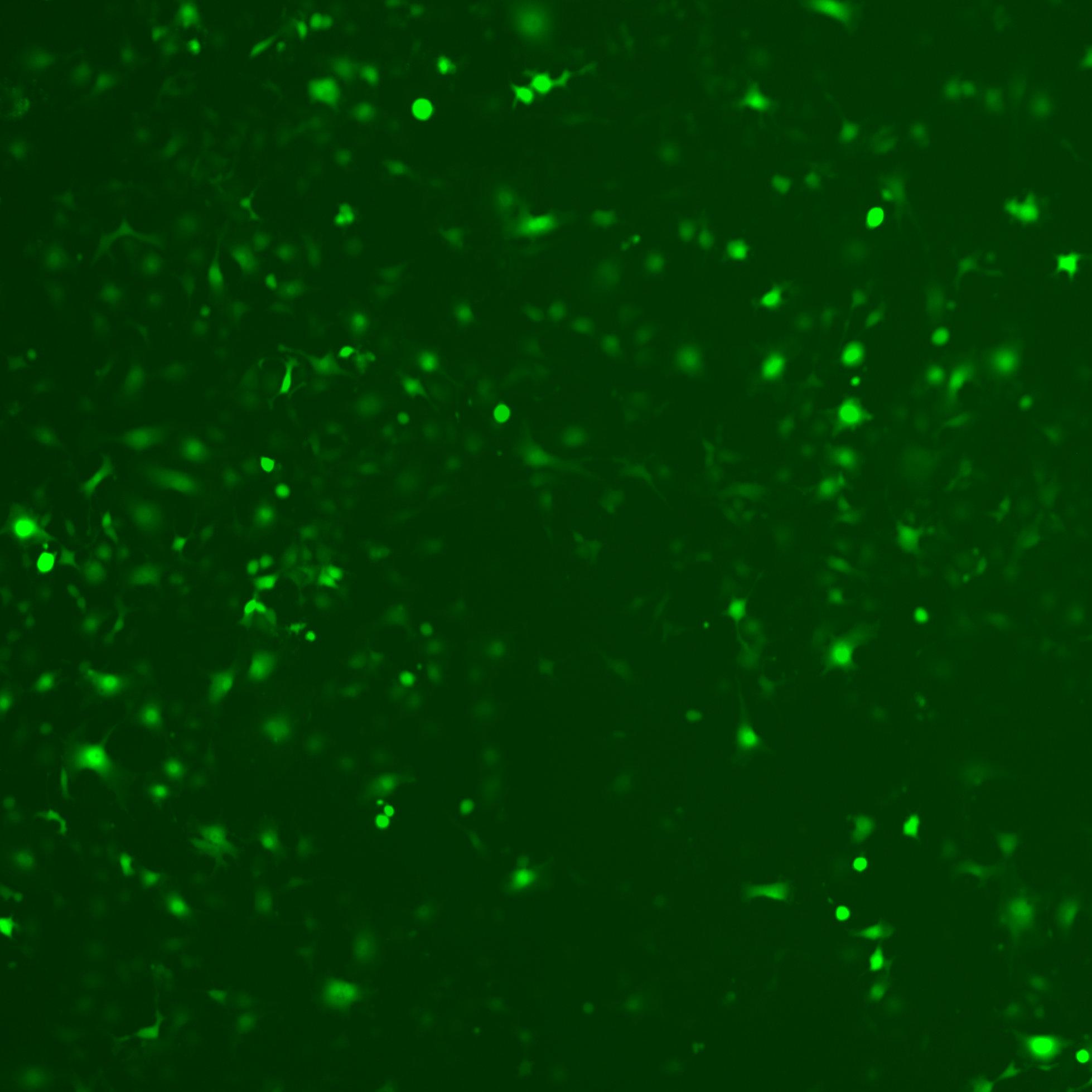

Supplement: Supplemental Information 12 [file peerj-10-12832-s012.zip › Original images 4 cell cultures/Figure1C-shDIDO1-day5.jpg]

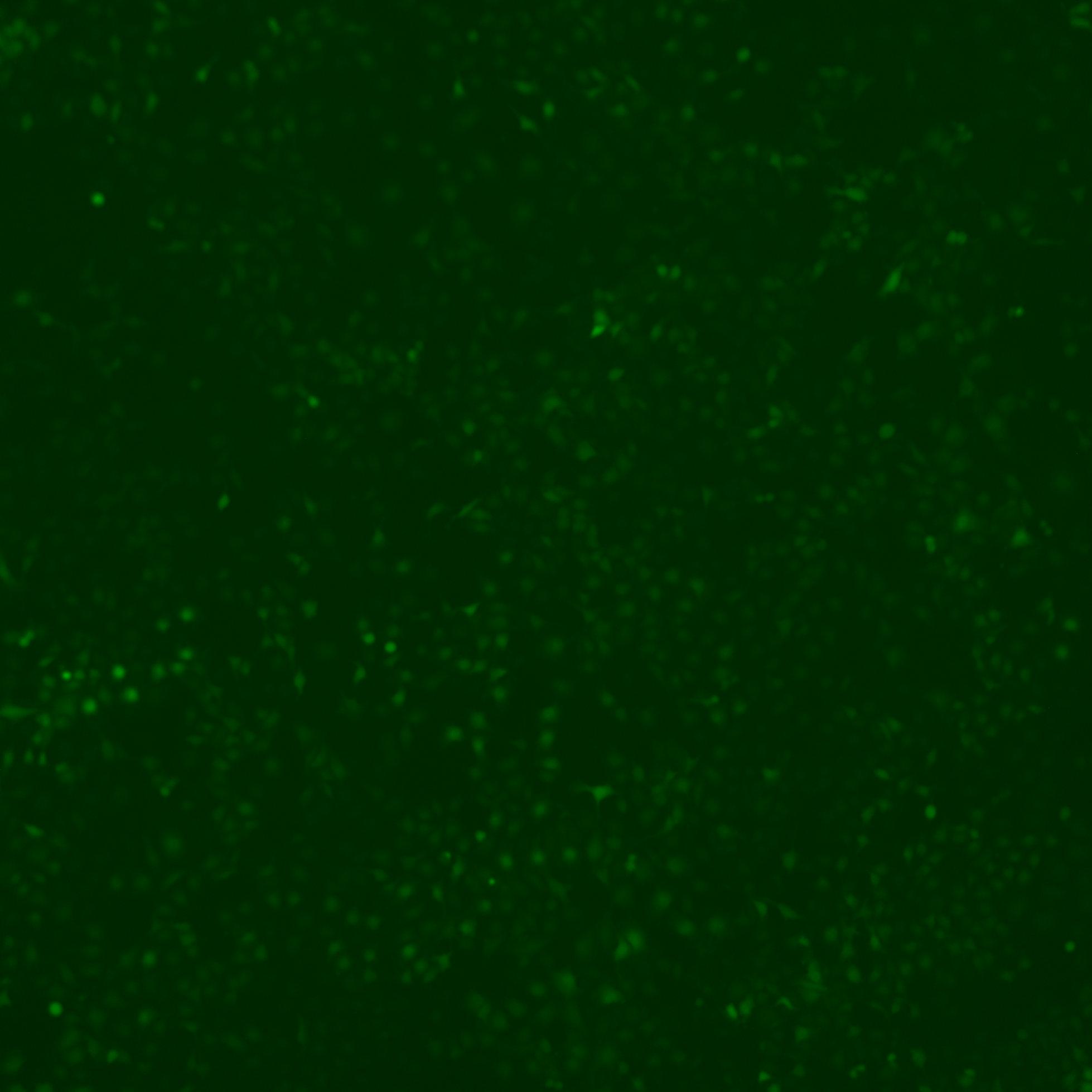

Supplement: Supplemental Information 12 [file peerj-10-12832-s012.zip › Original images 4 cell cultures/Figure1C-shCtrl-day4.jpg]

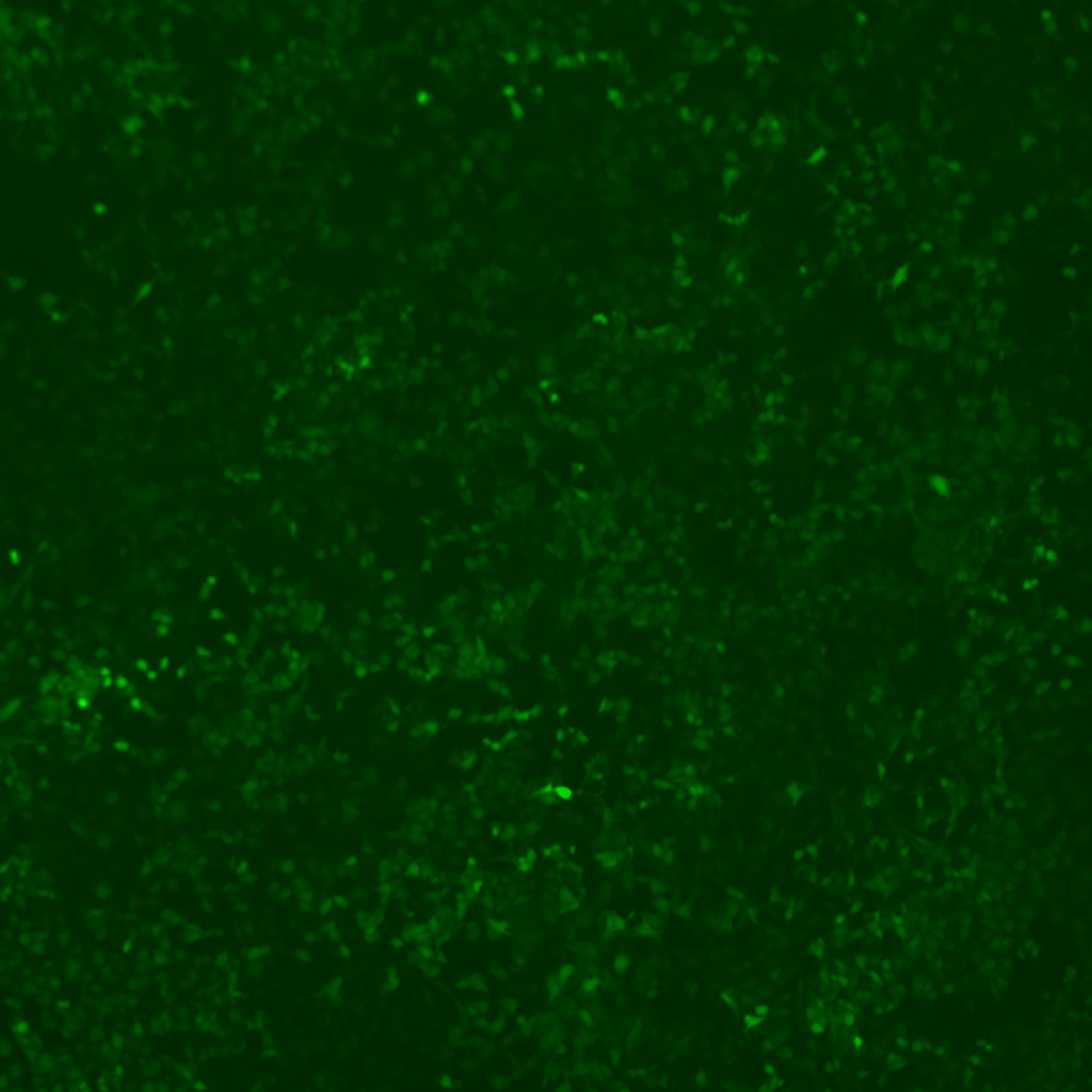

Supplement: Supplemental Information 12 [file peerj-10-12832-s012.zip › Original images 4 cell cultures/Figure1C-shCtrl-day5.jpg]

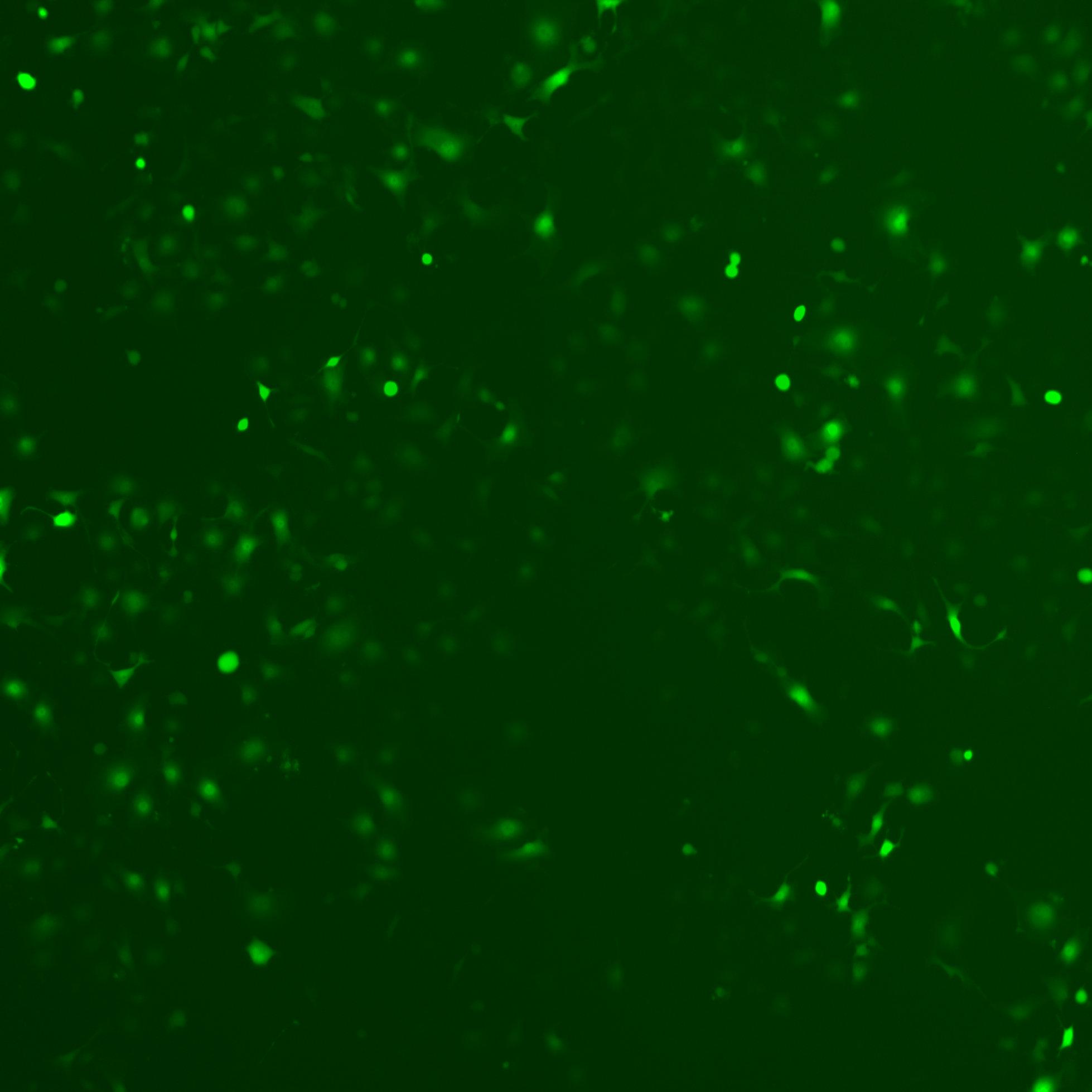

Supplement: Supplemental Information 12 [file peerj-10-12832-s012.zip › Original images 4 cell cultures/Figure1C-shDIDO1-day4.jpg]
