# Supplementary material for: In leukemia, knock-down of the death inducer-obliterator gene would inhibit the proliferation of endothelial cells by inhibiting the expression of CDK6 and CCND1
Source: PeerJ. 2022 Feb 1;10:e12832. doi: 10.7717/peerj.12832 (PMC8815367; doi:10.7717/peerj.12832)
Supplement: Supplemental Information 5 [file peerj-10-12832-s005.doc]

Supplementary table 1 The primers used for QRT-PCR

| **Gene symbol** | **F (5’-3’)** | **R (5’-3’)** | **Length (bp)** |
| --- | --- | --- | --- |
| GAPDH | TGACTTCAACAGCGACACCCA | CACCCTGTTGCTGTAGCCAAA | 121 |
| EPHA2 | AGAGGCTGAGCGTATCTTCATT | CGACTCGGCATAGTAGAGGTTG | 112 |
| NR4A2 | CGAACCCTGACTATCAAAT | GTTGGACCTGTATGCTAATC | 203 |
| CSF2 | CGGAAACTTCCTGTGCAA | CCAGCAGTCAAAGGGGAT | 92 |
| MET | AGTCATAGGAAGAGGGCATT | CTTCACTTCGCAGGCAGA | 206 |
| FOXM1 | GCAGCGACAGGTTAAGGTTGAG | GTTGTGGCGGATGGAGTTCTTC | 226 |
| EEF1A1 | CCCAGGACACAGAGACTTTATC | CAACACCAGCAGCAACAATC | 86 |
| FOXO3 | ACCTGATACCTGTTACCAAAGC | CAGAGGAAGCAATAAAGGAAACC | 140 |
| YWHAZ | AGCCATTGCTGAACTTGATACA | AATTTTCCCCTCCTTCTCCTG | 143 |
| VEGFC | AGGCTGGCAACATAACAGAGA | TCCCCACATCTATACACACCTC | 161 |
| RPS6KA1 | CCAGCACCCCAACATCATCAC | TTCCCGGACTCGTCCACATAC | 246 |
| EGFR | GGTGACCGTTTGGGAGTT | CCTGAATGACAAGGTAGCG | 245 |
| WNT5A | TCGACTATGGCTACCGCTTTG | CACTCTCGTAGGAGCCCTTG | 84 |
| RUNX2 | GGAATGCCTCTGCTGTTATG | GGATTTGTGAAGACGGTTATGG | 115 |
| HMGB1 | TATGGCAAAAGCGGACAAGG | CTTCGCAACATCACCAATGGA | 196 |
| PTGS2 | CTCCTGTGCCTGATGATTGC | CAGCCCGTTGGTGAAAGC | 215 |
| BTG2 | AGGCACTCACAGAGCACTACAA | CTCCTCGTACAAGACGCAGATG | 245 |
| JAG1 | GTCCATGCAGAACGTGAACG | GCGGGACTGATACTCCTTGA | 136 |
| SKP2 | AAGAGGAGCCCGACAGTGAGA | GGAGGCACAGACAGGAAAAGAT | 216 |
| TLR2 | CGGAAGATAATGAACACCAAGAC | AGATCCCAACTAGACAAAGACTG | 139 |
| CDK6 | CCTTAGCACAGCACCAC | GGGATTTCTCAGCCAGT | 167 |
| NRG1 | CGAAAGCCACTCTGTAATC | AGTCTCGGTAGGAATCAGG | 158 |
| MKI67 | GGAACAGCCTCAACCATCAG | CCACTCTTTCTCCCTCCTCTC | 210 |
| PRKCE | GAGCCTCGTTCACGGTTCTAT | GTAGTCAGGAGTCCCACAGAA | 204 |
| ATF3 | GCTAAGCAGTCGTGGTATG | CTGGAGTTGAGGCAAAGAT | 225 |
| NGF | CAGTTTTACCAAGGGAGCAG | TGTCAAGGGAATGCTGAAGT | 191 |
| CCND1 | GGTGGCAAGAGTGTGGAG | CCTGGAAGTCAACGGTAGC | 148 |
| PIM1 | AGATATTCCTTTCGAGCATG | AGGTGGATCTCAGCAGTTT | 198 |
| SRPK2 | AGAAGAGGCAGGCTGAGTT | TAGGGTCTATGTTCGCAAG | 300 |
| EIF4E | CTGCGGCTGATCTCCAAG | TTCCCACATAGGCTCAATACC | 132 |
| DIDO | CGCCAGCCTCACAACAAC | ATCAGCATCTCCAGGTCTCC | 219 |
