# Supplementary material for: In leukemia, knock-down of the death inducer-obliterator gene would inhibit the proliferation of endothelial cells by inhibiting the expression of CDK6 and CCND1
Source: PeerJ. 2022 Feb 1;10:e12832. doi: 10.7717/peerj.12832 (PMC8815367; doi:10.7717/peerj.12832)
Supplement: Supplemental Information 14 [file peerj-10-12832-s014.zip › Miame Checklist.docx]

**MIAME Checklist**

Part 1 Experiment description

*“The minimal information required in this section includes the type of the experiment (such as normal-versus-diseased comparison, time course, dose response, and so on) and the experimental variables, including parameters or conditions tested (such as time, dose, genetic variation or response to a treatment or compound).”*

*“this section specifies the experimental relationships between the array and sample entities—that is, which samples and which arrays were used in each hybridization assay. Each of these will be assigned unique identifiers that are cross-referenced with the information provided in the following sections.”*

- **cell lines**

The cell lines were maintained using Roswell Park Memorial Institute (RPMI) 1640 medium supplemented with 10% FBS and 1% penicillin/streptomycin at 37°C in a humidified atmosphere containing 5% CO2.

- experimental variables (HUVEC cell lines vs. K562-HUVEC co-cultivated cell lines, shDIDO1 cell lines vs. controls (shCtrl) cell lines)

- n-count : 14 samples
- tissues used for slide: cell lines

Part 2 Array design.

*“The aim of this section is to provide a systematic definition of all arrays used in the experiment, including the genes represented and their physical layout on the array.”*

*“The array-type definition includes information common to all arrays of a particular type (such as glass-slide spotted with PCR-amplified cDNA clones) as well as precise descriptions of the physical content of each element (spot or feature). This section consists of three parts: (i) a description of the array as a whole (such as platform type, provider and surface type); (ii) a description of each type of element or spot used (properties that are typically common to many elements, such as 'synthesized oligo-nucleotides' or 'PCR products from cDNA clones'); and (iii) a description of the specific properties of each element, such as the DNA sequence and, possibly, quality-control indicators.”*

- **Array series:** Human GeneChip primeview (Affymetrix, 901838)

- **Array type (human)**

We analyzed the GeneChip expression profiles of human umbilical vein endothelial cells transfected by RNAi lentiviral vectors, shDIDO1 cell line and shCtrl cell lines

Part 3 Samples

*“The MIAME 'sample' concept represents the biological material (or biomaterial) for which the gene expression profile is being established. This section is divided into three parts which describe the source of the original sample (such as organism taxonomy and cell type) and any biological*in vivo*or*in vitro*treatments applied, the technical extraction of the nucleic acids, and their subsequent labeling.”*

-         **Cy3/Cy5 labels for tissues:** Biotin

-         **Labelling protocol used:** Biotinylated cRNA were prepared according to the standard Affymetrix protocol from 50-500ng total RNA (Expression Analysis Technical Manual, 2001, Affymetrix).

-         **Sample extraction protocol used :** Trizol extraction of total RNA was performed according to the manufacturer's instructions.

-         **Amount of sample labelled:** 14 samples

Part 4 Hybridizations

*“This section defines the laboratory conditions under which the hybridizations were carried out. Other than a free-text description of the hybridization protocol, MIAME requires that a number of critical hybridization parameters are explicitly specified: choice of hybridization solution (such as salt and detergent concentrations), nature of the blocking agent, wash procedure, quantity of labeled target used, hybridization time, volume, temperature and descriptions of the hybridization instruments.”*

-         **Hybridization protocol:**

Following fragmentation, cRNA were hybridized for 16-18 hr at 45 ℃ on GeneChip PrimeView Human Gene Expression Array. GeneChips were washed and stained in the Affymetrix Fluidics Station 450.

GeneChips were scanned using the GeneChip Scanner 3000.

Part 5 Measurements

*“Image data should be provided as raw scanner image files (such as TIFF), accompanied by scanning information that includes relevant scan parameters and laboratory protocols.”*

-         **Which version of scanner software used**

-         **Laser power for scan**

-         **Instrument model numbers**

-         **Must save original .tiff format images (composite image is optional)**

*For each experimental image, a microarray quantification matrix contains the complete image analysis output as directly generated by the image analysis software (normally provided as separate spreadsheet-type files). Note that for a given image this is a 2D matrix, where array elements (spots or features) constitute one dimension and quantification types (such as mean and median intensity, mean or median background intensity) are the second dimension.*

----

The human GeneChip microarrays (901838, Affymetrix) were hybridized with samples to determine gene expression profiles according to the manufacturer’s instructions. The background correction, quantile normalization and probe summarization of the microarray data were performed using the Robust Multiarray average algorithm to obtain the gene expression matrix.

*Finally, the gene expression matrix (summarized information) consists of sets of gene expression levels for each sample. If microarray quantification matrices can be considered spot/image centric, then the gene expression matrix is gene/sample centric. At this point, the expression values may have been normalized, consolidated and transformed in any number of ways by the submitter in order to present the data in a form amenable to scientific analysis. Rather than attempting to impose a standard for gene expression values, MIAME indicates preferred detailed specifications of all numerical calculations applied to unprocessed quantifications in (b) that have led to the data in (c). Experimenters are encouraged, though not required, to provide reliability indicators (such as s.d.) for each data point.*

*-----* The cut-off for the background correction was 20%, and the coefficient of variation was 25%. The Benjamini-Hochberg method was used to correct the significant difference level (FDR). The screening criteria for significantly different geneswere: |Fold Change|>1.5 and FDR<0.05.

Part 6 Normalization controls

*“A typical microarray experiment involves a number of hybridization assays in which the data from multiple samples are analyzed to identify relative changes in expression levels, identify differentially expressed genes and, in many cases, discover classes of genes or samples having similar patterns of expression.”*
